# Mouse sperm energy restriction and recovery (SER) revealed novel metabolic pathways

**DOI:** 10.3389/fcell.2023.1234221

**Published:** 2023-08-15

**Authors:** Ana Romarowski, Jasna Fejzo, Saman Nayyab, David Martin-Hidalgo, Maria G. Gervasi, Melanie Balbach, Sara Violante, Ana M. Salicioni, Justin Cross, Lonny R. Levin, Jochen Buck, Pablo E. Visconti

**Affiliations:** ^1^ Department of Veterinary and Animal Sciences, University of Massachusetts, Amherst, MA, United States; ^2^ Instituto de Biología y Medicina Experimental, Consejo Nacional de Investigaciones Científicas y Técnicas (IBYME-CONICET), Buenos Aires, Argentina; ^3^ Institute for Applied Life Sciences, University of Massachusetts, Amherst, MA, United States; ^4^ Hospital San Pedro de Alcántara, Cáceres, Spain; ^5^ Department of Pharmacology, Weill Cornell Medicine, New York, NY, United States; ^6^ Memorial Sloan Kettering Cancer Center, New York, NY, United States

**Keywords:** sperm, assisted reproductive technologies, metabolism, AMP, ATP, citrate, L-carnitine

## Abstract

Mammalian sperm must undergo capacitation to become fertilization-competent. While working on mice, we recently developed a new methodology for treating sperm *in vitro,* which results in higher rates of fertilization and embryo development after *in vitro* fertilization. Sperm incubated in media devoid of nutrients lose motility, although they remain viable. Upon re-adding energy substrates, sperm resume motility and become capacitated with improved functionality. Here, we explore how sperm energy restriction and recovery (SER) treatment affects sperm metabolism and capacitation-associated signaling. Using extracellular flux analysis and metabolite profiling and tracing via nuclear magnetic resonance (NMR) and mass spectrometry (MS), we found that the levels of many metabolites were altered during the starvation phase of SER. Of particular interest, two metabolites, AMP and L-carnitine, were significantly increased in energy-restricted sperm. Upon re-addition of glucose and initiation of capacitation, most metabolite levels recovered and closely mimic the levels observed in capacitating sperm that have not undergone starvation. In both control and SER-treated sperm, incubation under capacitating conditions upregulated glycolysis and oxidative phosphorylation. However, ATP levels were diminished, presumably reflecting the increased energy consumption during capacitation. Flux data following the fate of ^13^C glucose indicate that, similar to other cells with high glucose consumption rates, pyruvate is converted into ^13^C-lactate and, with lower efficiency, into ^13^C-acetate, which are then released into the incubation media. Furthermore, our metabolic flux data show that exogenously supplied glucose is converted into citrate, providing evidence that in sperm cells, as in somatic cells, glycolytic products can be converted into Krebs cycle metabolites.

## Introduction

After leaving the testis, mammalian sperm undergo two post-testicular maturation processes, one in the male epididymis, known as epididymal maturation (Gervasi and Visconti, 2017), and the second in the female genital tract, known as capacitation (Austin, 1951; Chang, 1951). Contrary to epididymal maturation, capacitation can be mimicked *in vitro* in a defined medium. While there are some species-specific variations, mammalian sperm capacitation media are isotonic physiological solutions containing bicarbonate (HCO_3_^−^), calcium (Ca^2+^), a protein source that is usually serum albumin, and energy substrates. Functionally, capacitation results in sperm changing their motility pattern (e.g., hyperactivation) (Ho and Suarez, 2001) and being able to undergo a physiologically induced acrosome reaction. At the molecular level, capacitation is initiated as soon as sperm are exposed to HCO_3_^−^ in the seminal plasma or *in vitro* capacitation media. HCO_3_^−^ stimulates the activity of soluble adenylyl cyclase ADCY10 (aka sAC) with the consequent increase in cAMP and protein kinase A (PKA) activity (Gervasi and Visconti, 2016). Activation of this pathway occurs in less than 1 min and triggers signaling cascades that include increases in intracellular pH, intracellular Ca^2+^, protein tyrosine phosphorylation, and hyperpolarization of the sperm plasma membrane potential. These sperm signaling pathways elicit processes with increased energy demand. In capacitating sperm, most of the energy consumed is used for movement mediated by dynein ATPases; however, many other functions also require ATP, including ion homeostasis, enzymatic reactions, and exocytotic events.

Like somatic cells, spermatozoa harbor the molecular machinery for glycolysis and oxidative phosphorylation. However, contrary to other cell types, these two metabolic pathways are postulated to be physically separated in different sperm compartments; glycolytic enzymes are most abundantly found in the principal piece, while mitochondria and oxidative phosphorylation (OxPhos) machinery are found exclusively in the mid-piece (Visconti, 2012; Ferramosca and Zara, 2014; Amaral, 2022). We recently used extracellular flux analysis to show that, during capacitation, mouse sperm incubated only in the presence of glucose upregulated both glycolysis and OxPhos (Balbach et al., 2020b). Under these conditions, inhibitors of glycolysis prevented the stimulation of OxPhos, suggesting that these metabolic pathways are coupled in sperm (Balbach et al., 2020b) as they are in somatic cells.

During our investigations of sperm metabolism, we explored the effects of nutrient starvation. When cauda epididymal mouse sperm are incubated in nutrient-free media, their motility ceases within 30–40 min. These “starved” sperm remain viable, and when they are allowed to capacitate in the presence of restored nutrients (i.e., glucose and/or pyruvate), the “recovered” sperm show improved functionality relative to sperm directly capacitated after collection from the epididymis. We refer to this procedure as sperm energy restriction and recovery (SER), and relative to untreated sperm, a higher percentage of SER-treated sperm undergo hyperactivation and more efficiently fertilized metaphase II-arrested oocytes after *in vitro* fertilization (IVF). More surprisingly, zygotes derived from SER-treated sperm had an increased probability to develop into blastocysts, and when transferred into pseudo-pregnant females, blastocysts derived from SER treatment were three times more likely to successfully develop into pups (Navarrete et al., 2019). SER treatment also increased cleavage and blastocyst development in bovines after intracytoplasmic sperm injection (Navarrete et al., 2019), as well as the obtention of ram blastocysts after IVF (Al-Hafedh and Cedden, 2023).

In this work, we used metabolic flux assays, along with sperm metabolite profiling and tracing, to investigate the metabolic consequences of starvation and subsequent recovery. The most significant differences from normally capacitated sperm we observed were that starved sperm have significantly increased levels of AMP and L-carnitine. Both metabolites recovered to pre-starvation levels during rescue. Regardless of the sperm treatment, capacitation conditions increased glycolysis and OxPhos. However, despite this upregulation of energy pathways, capacitated sperm have reduced ATP levels, suggesting increased energy consumption. Finally, using NMR, we observed the conversion of ^13^C-glucose into ^13^C-citrate, indicating that glycolysis has the potential to couple with the mitochondrial Krebs cycle.

## Results

### Phosphorylation pathways are blocked under starvation conditions and recovered upon rescue

**FIGURE 1:**
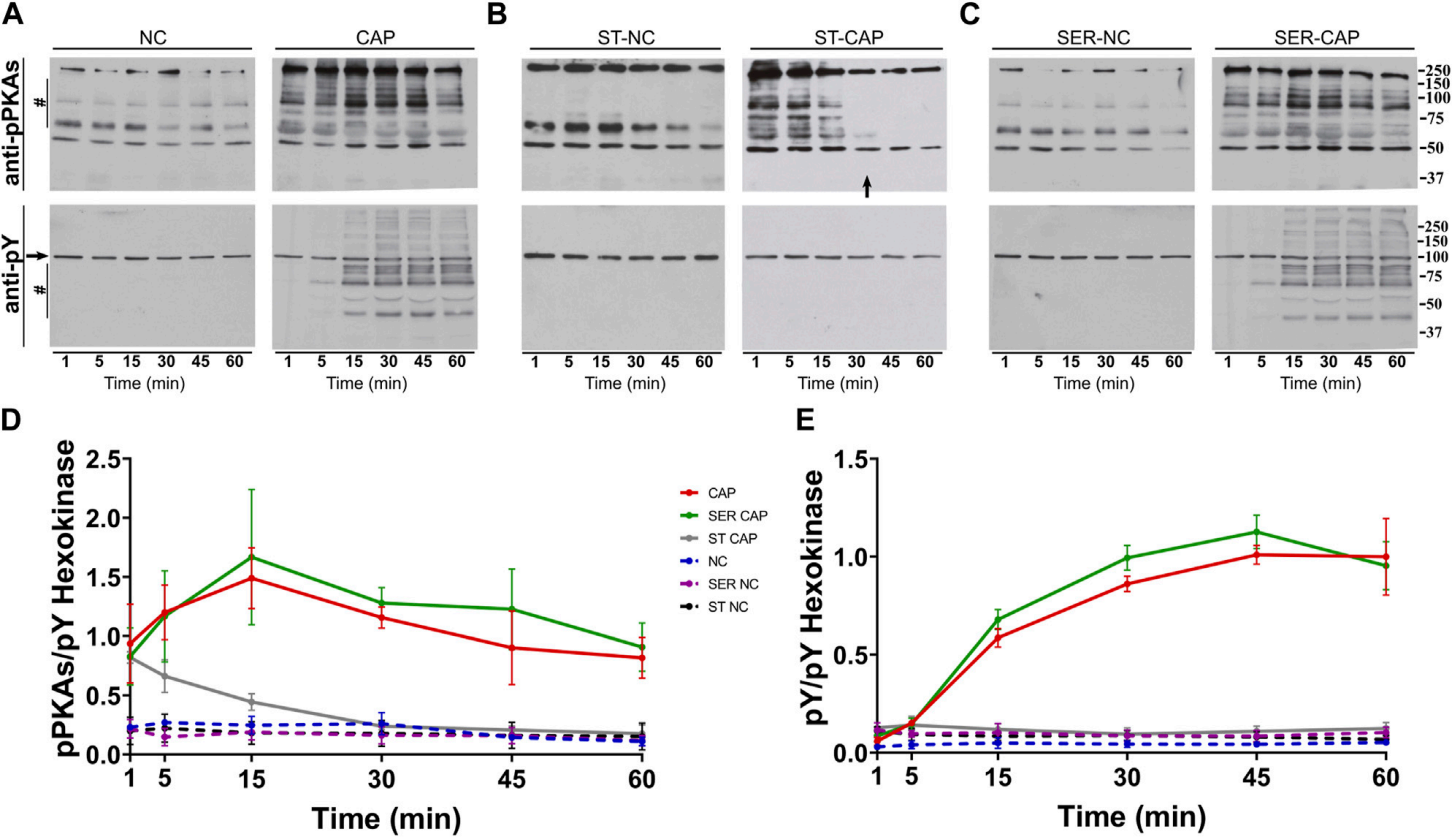
SER treatment recovers the activation of the PKA/pY pathway. Sperm were incubated under the following different conditions: **(A)** continuously incubated with glucose and pyruvate under the NC (without BSA and without HCO_3_^−^) or CAP (with BSA and HCO_3_^−^) condition; **(B)** continuously incubated without glucose and pyruvate under the NC (ST-NC) or CAP (ST-CAP) condition; or **(C)** incubated without glucose and pyruvate under the NC condition until motility stopped (∼30–40 min) and recovered with glucose and pyruvate in non-capacitated medium (SER-NC) or in capacitated medium (SER-CAP). At the indicated time points, proteins were extracted and separated by 8% SDS-PAGE and immunoblotted using anti-phosphorylated PKA substrates (pPKAs) and anti-phospho-tyrosine (pY) antibodies. Equal loading was visualized by tyrosine-phosphorylated hexokinase. Representative images are shown. The arrow in (B) ST-CAP indicates that PKA substrate dephosphorylation occurs at the time when sperm stop moving. **(D–E)** The respective quantitative analysis was performed by measuring the optical density of the bands in the region marked by # and normalized using tyrosine-phosphorylated hexokinase as the control. The results are expressed as the mean ± SEM of at least three independent experiments.

We used Western blotting with anti-phospho-PKA substrates (anti-pPKAs) (Figures 1A–C, upper panels) and anti-phosphotyrosine (anti-pY) (Figures 1A–C, lower panels) antibodies to explore the effect of SER on capacitation-dependent protein phosphorylation signaling cascades. As expected, in non-capacitating (NC) media, there were no significant changes in the PKA substrates or pY phosphorylation patterns under any condition (Figures 1A–C, NC), whereas in CAP media, PKA phosphorylation of substrates increased in less than 1 min (Figure 1A, CAP, upper panel) and that of pY increased after 15 min of capacitation (Figure 1A, CAP, lower panel). Under starving conditions, PKA phosphorylation of substrates was initially stimulated, followed by dephosphorylation at 30 min, which coincides with the time point when sperm stop moving (see arrow in Figure 1B). In the absence of nutrients, whether under NC or CAP conditions, pY did not change. Importantly, upon recovery (i.e., when glucose and pyruvate are added back) under conditions that support capacitation, phosphorylation of PKA substrates and tyrosine residues recovered (Figure 1C, SER-CAP). Densitometric analysis confirmed that PKA substrates and pY phosphorylation patterns after recovery, under either NC or CAP conditions, are indistinguishable from their respective controls persistently incubated in the presence of nutrients (Figures 1D, E).

### Glucose alone is sufficient for sperm capacitation and to maintain glycolysis and oxidative phosphorylation pathways

**FIGURE 2:**
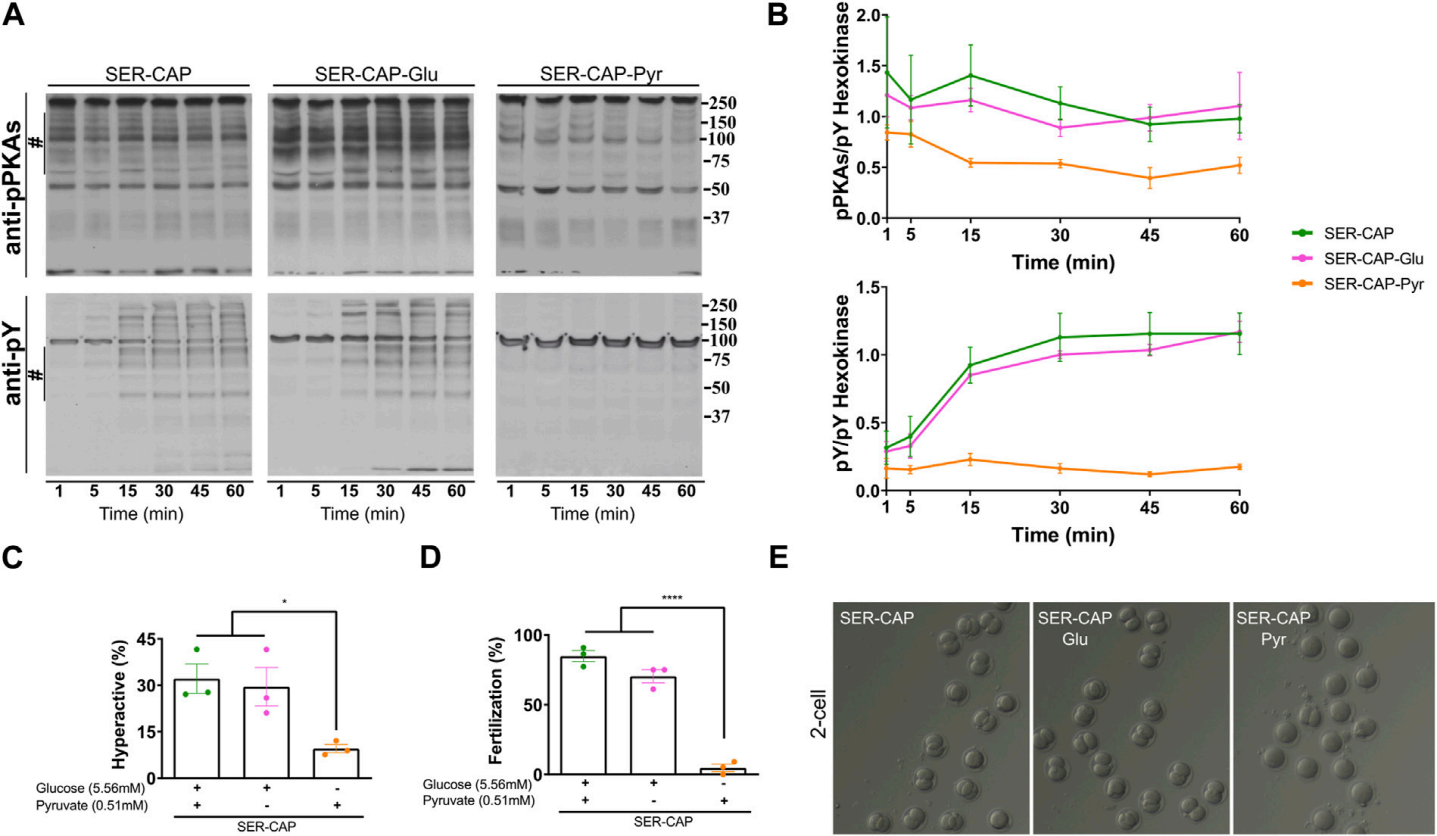
Glucose is sufficient to induce the activation of the PKA/pY pathway and the increased hyperactivation and fertilization rates obtained after SER. **(A)** Sperm were incubated without glucose and pyruvate under the NC condition until motility stopped (∼30–40 min) and recovered with glucose and pyruvate in capacitated medium (SER-CAP) or with glucose only in capacitated medium (SER-CAP-Glu) or with pyruvate only in capacitated medium (SER-CAP-Pyr). At the indicated time points, proteins were extracted and separated by 8% SDS-PAGE and immunoblotted using anti-phosphorylated PKA substrates (pPKAs) and anti-phospho-tyrosine (pY) antibodies. As a loading control, hexokinase was used. Representative images are shown. **(B)** The respective quantitative analysis was performed by measuring the optical density of the bands in the region marked by # and normalized using tyrosine-phosphorylated hexokinase as the control. The results are expressed as the mean ± SEM of at least three independent experiments. **(C)** Percentage of hyperactivated out of the total motile sperm after 1 h incubation under the different conditions. The results are expressed as the mean ± SEM of three independent experiments. ANOVA with Tukey’s multiple comparison test was performed. * indicates a significant difference with *p* < 0.05. **(D)** Percentage of fertilization indicated by oocytes that reached the two-cell embryo stage. The results are expressed as the mean ± SEM of three independent experiments. ANOVA with Tukey’s multiple comparison test was performed. **** indicates a significant difference with *p* < 0.0001. **(E)** Representative images of two-cell embryos obtained after IVF with CD-1 female oocytes and CD1 male sperm, SER-treated recovered with glucose and pyruvate or SER-treated recovered with glucose only or SER-treated recovered with pyruvate only.

Using glucose alone in the recovery step, we observed similar PKA substrates and pY phosphorylation patterns as when using glucose and pyruvate combined (Figures 2A, B); thus, glucose alone was sufficient to recover capacitation-induced changes in PKA and tyrosine phosphorylation. In contrast, pyruvate alone was only partially able to recover phosphorylation of PKA substrates and did not support the capacitation-induced increase in pY. In addition to phosphorylation, capacitation is accompanied by changes in the sperm motility pattern, known as hyperactivation. Recovery with glucose, but not pyruvate, was sufficient to increase the percentage of hyperactivated sperm to the same level as observed for media containing glucose and pyruvate (Figure 2C). Finally, assessing the definitive readout of capacitation, i.e., the ability to fertilize an oocyte, glucose, but not pyruvate, supported the fertilizing capacity of SER sperm to levels comparable to those obtained in media containing glucose and pyruvate (Figures 2D, E). Altogether, these data support the use of glucose alone to explore how metabolite levels are altered during capacitation and SER treatments.

**FIGURE 3:**
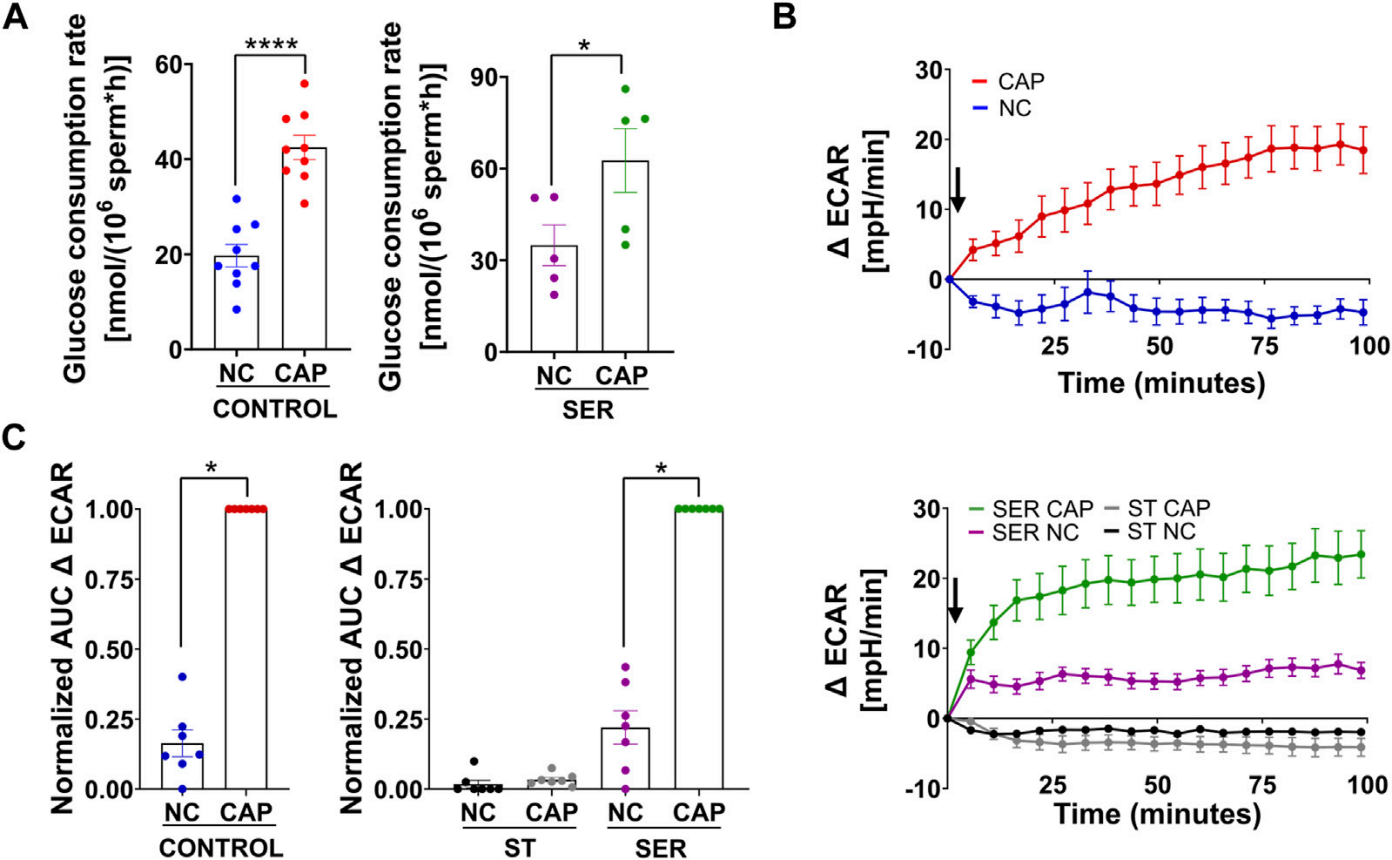
Glucose alone is sufficient to maintain glycolysis pathways. **(A)** The glucose consumption rate was determined using the Amplex™ Red Glucose/Glucose Oxidase Assay Kit. Sperm were subjected to different incubation conditions as follows: continuously incubated with glucose in NC (without BSA and without HCO_3_^−^) or CAP (with BSA and HCO_3_^−^); incubated without glucose under the NC condition until motility stopped (∼30–40 min) and recovered with glucose in non-capacitated medium (SER-NC) or in capacitated medium (SER-CAP). The results are expressed as the mean ± SEM of at least five independent experiments. T-tests between NC and CAP (control and SER) conditions were performed. **** indicates a significant difference with *p* < 0.0001; * indicates a significant difference with *p* < 0.05. **(B)** Measurement of the extracellular acidification rate (ECAR) by Seahorse. Upper panel: the arrow indicates the release of the content of port B (5.6 mM glucose TYH medium with DMSO (vehicle) for wells with NC sperm; 5.6 mM glucose TYH medium with 10 mM dbcAMP and 1 mM IBMX for wells with CAP sperm). Lower panel: the arrow indicates the release of the content of port B (starving TYH medium with DMSO (vehicle) for wells with ST NC sperm; starving TYH medium with 10 mM dbcAMP and 1 mM IBMX for wells with ST CAP sperm; 56 mM glucose TYH medium with DMSO (vehicle) for wells with SER NC sperm; 56 mM glucose TYH medium with 10 mM dbcAMP and 1 mM IBMX for wells with SER CAP sperm). **(C)** Area under the curve (AUC) of ECAR–ECAR at time 0 min for each time point (=Δ ECAR), normalized to CONTROL CAP (left panel) or to SER CAP (right panel). The results are expressed as the mean ± SEM of seven independent experiments. T-tests with Wilcoxon’s matched-pair signed rank test between NC and CAP (control, ST, and SER) conditions were performed. * indicates a significant difference with *p* < 0.05.

**FIGURE 4:**
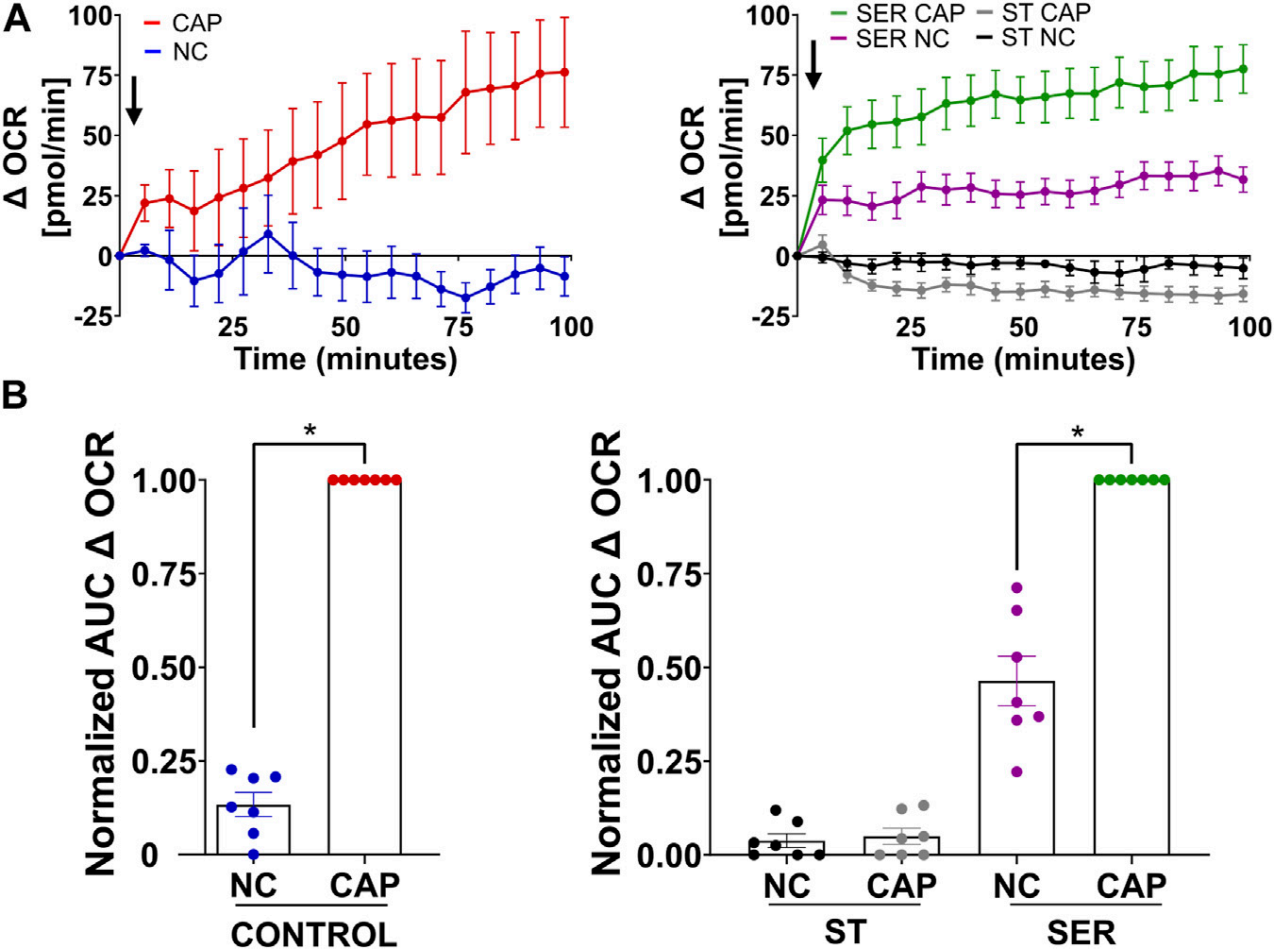
Glucose alone is sufficient to maintain oxidative phosphorylation pathways. **(A)** Measurement of the oxygen consumption rate (OCR) by Seahorse under the following incubation conditions: continuously incubated with glucose in NC (without BSA and without HCO_3_^−^) or CAP (with dbcAMP and IBMX); continuously incubated without glucose in NC (ST-NC) or CAP (ST-CAP); incubated without glucose under NC condition until motility stopped (∼30–40 min) and recovered with glucose in non-capacitated medium (SER-NC) or in capacitated medium (SER-CAP). Left panel: the arrow indicates the release of the content of port B (5.6 mM glucose TYH medium with DMSO (vehicle) for wells with NC sperm; 5.6 mM glucose TYH medium with 10 mM dbcAMP and 1 mM IBMX for wells with CAP sperm). Right panel: the arrow indicates the release of the content of port B (starving TYH medium with DMSO (vehicle) for wells with ST NC sperm; starving TYH medium with 10 mM dbcAMP and 1 mM IBMX for wells with ST CAP sperm; 56 mM glucose TYH medium with DMSO (vehicle) for wells with SER NC sperm; 56 mM glucose TYH medium with 10 mM dbcAMP and 1 mM IBMX for wells with SER CAP sperm). **(B)** AUC of OCR–OCR at time 0 min for each time point (=Δ OCR), normalized to CONTROL CAP (left panel) or to SER CAP (right panel). The results are expressed as the mean ± SEM of seven independent experiments. T-tests with Wilcoxon’s matched-pair signed rank test between NC and CAP (control, ST and SER) conditions were performed. * indicates a significant difference with *p* < 0.05.

We previously showed that glucose consumption is increased in capacitating sperm ((Hidalgo et al., 2020) and Figure 3A, left panel), and both glycolysis and OxPhos are stimulated during capacitation (Balbach et al., 2020b). In SER-treated sperm (i.e., following nutrient deprivation), glucose consumption was also increased in CAP conditions (Figure 3A, right panel). Because glucose was measured using glucose oxidase coupling to hydrogen peroxide formation (H_2_O_2_), we should consider H_2_O_2_ is released by capacitated sperm (Takei et al., 2021), implying that glucose consumption might be slightly higher than that shown in Figure 3A. Overall, this observation does not change our main conclusion regarding the increase in glucose consumption during capacitation. Consistently, the capacitation-associated increase in glucose consumption was accompanied by upregulation of glycolysis. We used an extracellular flux analyzer to measure real-time changes in proton release (ECAR) and oxygen consumption rate (OCR) (Ferrick et al., 2008). While ECAR and OCR serve as readouts of the changes in the rate of glycolysis and oxidative phosphorylation, respectively, proton release and oxygen consumption may occur via other metabolic pathways. We have previously shown that the capacitation-induced changes in ECAR and OCR in mouse sperm are completely inhibited upon addition of 2-deoxyglucose (inhibits the first step of glycolysis) or antimycin A and rotenone (inhibits complex III and complex I of the electron transport chain, respectively), respectively (Balbach et al., 2020b; Balbach et al., 2020a), implying that the Seahorse measurements reflect glycolysis by proton release and OxPhos by oxygen consumption. As anticipated, under starvation conditions, the ECAR (Figures 3B, C, lower panel and right panel; Supplementary Figure 1A, right panel) and OCR (Figures 4A, B, right panel and right panel; Supplementary Figure 1B, right panel) decreased to almost undetectable levels. Interestingly, the rates of glycolysis and OxPhos transiently increased when starved sperm were placed in capacitating media (Supplementary Figures 1A, B, right panels). Presumably, the capacitation-induced increases in glycolysis and OxPhos were initiated but could not be sustained in the absence of exogenous nutrients. When glucose was added back to starved sperm, the increase in the ECAR (Figures 3B, C lower panel and right panel; Supplementary Figure 1A, right panel) and OCR (Figure 4A, right panel; B, right panel; and Supplementary Figure 1B, right panel) was rescued, and this recovery was higher under capacitating conditions as observed in control conditions. It appears that the ECAR (Figure 3B, lower panel vs. upper panel) and OCR (Figure 4A, right panel vs. left panel) in SER-treated sperm have faster kinetics than those of sperm persistently incubated in the presence of nutrients. However, it is important to be careful when comparing these samples because in control sperm, glucose was present from the beginning of the measurements, while in SER-treated sperm, glucose was added to starved sperm during the measurements. Overall, these results are consistent with those of our previous experiments demonstrating that a glycolytic substrate can support the increase in OCR (Balbach et al., 2020b), suggesting that sperm can couple glycolysis and mitochondrial OxPhos as observed in somatic cells.

### NMR and MS metabolomics studies revealed significant changes in sperm incubated under starvation conditions

**FIGURE 5:**
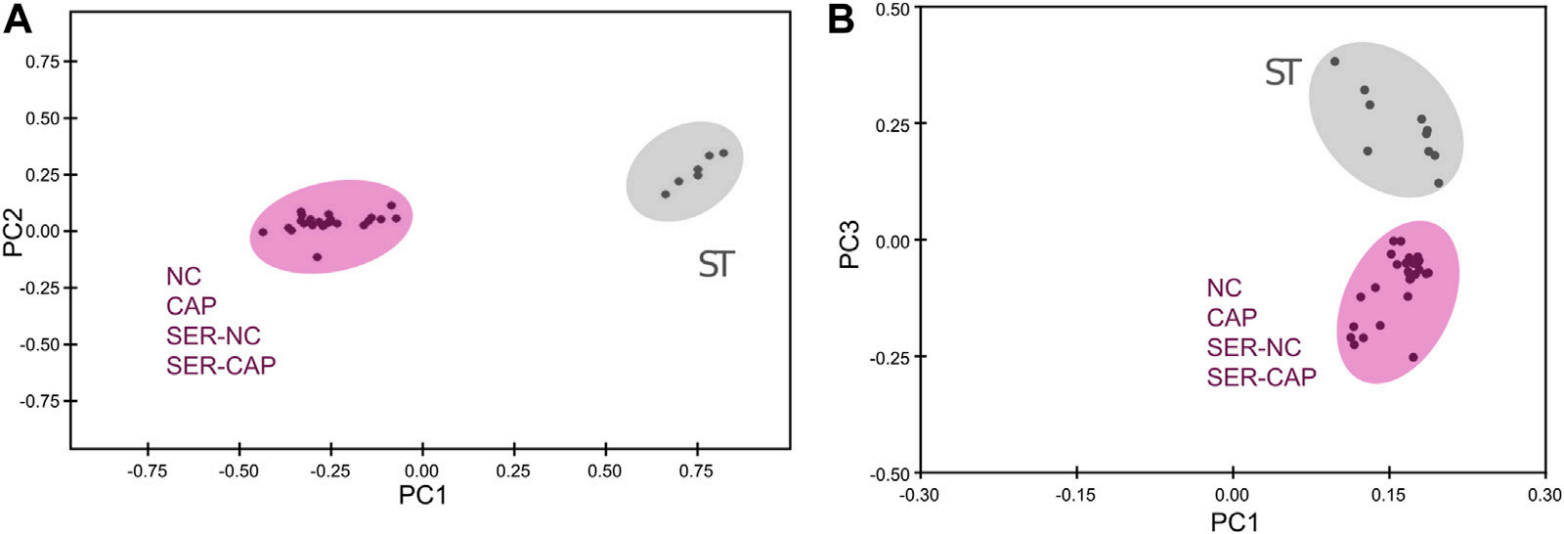
Significant metabolomic changes at the starving step. Measurement of metabolites by NMR and MS was performed after incubation under the following conditions: continuously incubated with glucose in NC (without BSA and without HCO_3_^−^) or CAP (with BSA and HCO_3_^−^); continuously incubated without glucose in NC (ST); incubated without glucose in NC condition until motility stopped (∼30–40 min) and recovered with glucose in non-capacitated medium (SER-NC) or in capacitated medium (SER-CAP). **(A)** PCA score plot of metabolite profiles generated by 1D ^1^H NMR spectra. ST sperm are surrounded by a gray circle (*N* = 6); NC, CAP, SER-NC, and SER-CAP are surrounded by a pink circle (*N* = 5) to show the different clusters. **(B)** PCA score plot of metabolite profiles generated by MS data. ST sperm are surrounded by a gray circle (*N* = 10); NC, CAP, SER-NC, and SER-CAP are surrounded by a pink circle (*N* = 7) to show the different clusters.

We used NMR and MS metabolomics profiling to assess changes in sperm metabolites during the different treatments. Metabolite profiles measured via NMR are summarized in Table 1, and metabolites identified via MS are listed in Table 2. Principal component analysis of the NMR spectra revealed that extracts from sperm incubated in the presence of glucose under any condition clustered together, while starved samples defined a distinct cluster (Figure 5A). Similar results were observed with MS data (Figure 5B). When starved sperm samples were excluded from PCA, metabolites from non-capacitated and capacitated sperm formed distinct clusters, regardless of when glucose was added (Supplementary Figure S2). Not surprisingly, glycolytic intermediates, which are significantly reduced upon starvation (Table 2), are recovered after rescue to levels comparable to those of their respective control (NC and CAP, respectively). Metabolic profiles of SER-NC and NC sperm, as well as those of SER-CAP and CAP sperm, are very similar. For example, in capacitating sperm, in both control and SER, cAMP is elevated (Table 2), consistent with what is known about signal transduction in capacitating sperm (Gervasi and Visconti, 2016) and the increase in phosphorylation of PKA substrates (Figure 1, Figures 2A, B). Thus, most changes in metabolites caused by starvation did not persist following glucose recovery, and the steady-state concentrations of metabolites depend more on the regulation of signaling pathways during capacitation than on whether the sperm recover from starvation.

**TABLE 1:** Sperm metabolites identified by NMR, indicating whether or not they were present in the supernatant of sperm samples. The type of the NMR experiment used for data analysis is indicated and explained in the *Methods* section.

Metabolite	Sperm	Supernatant	Experiment
Glucose	Yes	Yes	1H, TOCSY, HSQC
Lactic acid	Yes	Yes	1H, TOCSY, HSQC
Citric acid	Yes	No	1H, TOCSY, HSQC
Beta-nicotinamide adenine dinucleotide	Yes	No	1H, TOCSY
Adenosine 5-triphosphate	Yes	No	1H
Adenosine 5-diphosphate	Yes	No	1H
Adenosine 5-monophosphate	Yes	No	1H
L-Acetyl carnitine	Yes	No	1H, TOCSY, HSQC
L-Carnitine	Yes	No	1H, TOCSY, HSQC
Acetic acid	Yes	Yes	1H, TOCSY, HSQC

**TABLE 2:** Sperm metabolites identified by MS. Quadruplicate AUC values of each condition for each experiment were averaged, and all conditions values within the same experiment were normalized against the corresponding CAP value. Finally, the values were averaged across all the independent experiments.

	NC		CAP		ST-NC		ST-CAP		SER-NC		SER-CAP	
Metabolite	Mean	SEM	Mean	SEM	Mean	SEM	Mean	SEM	Mean	SEM	Mean	SEM
2-Phosphoglyceric acid	0.37	0.04	1	0	0.02	0.01	0.02	0.00	0.18	0.06	0.57	0.09
Adenine	1.46	0.25	1	0	1.63	0.30	0.85	0.30	1.38	0.25	1.22	0.16
Adenosine	1.62	0.49	1	0	0.92	0.34	2.82	1.18	1.38	0.24	1.04	0.18
Adenosine 3-5-cyclic monophosphate	0.31	0.04	1	0	0.35	0.16	0.21	0.03	0.36	0.17	0.79	0.17
Adenosine 5-diphosphate	3.49	0.79	1	0	2.98	1.55	1.26	0.21	3.82	1.59	1.55	0.41
Adenosine 5-monophosphate	1.83	0.19	1	0	13.60	4.52	8.80	1.89	2.05	0.28	1.22	0.15
Adenosine 5-triphosphate	2.44	0.36	1	0	0.28	0.10	0.07	0.01	2.06	0.55	0.72	0.07
alpha-D(+)Mannose 1-phosphate	0.61	0.20	1	0	0.05	0.02	0.04	0.02	0.51	0.22	0.93	0.18
alpha-D-Glucose-1-phosphate	0.68	0.06	1	0	0.04	0.01	0.03	0.01	0.62	0.10	0.70	0.08
Arabinose-5-phosphate	1.83	0.47	1	0	0.19	0.07	0.28	0.17	1.43	0.37	0.97	0.25
Beta-nicotinamide adenine dinucleotide	1.32	0.17	1	0	1.34	0.34	0.87	0.13	1.24	0.27	0.81	0.07
Citric acid	0.65	0.10	1	0	0.24	0.01	0.34	0.02	0.42	0.06	0.70	0.10
Cytidine	0.92	0.23	1	0	1.00	0.15	0.59	0.18	1.04	0.42	1.12	0.58
D-Fructose 6-phosphate	1.39	0.24	1	0	0.02	0.01	0.00	0.00	1.07	0.23	0.73	0.14
D-Glucose 6-phosphate	1.35	0.23	1	0	0.01	0.00	0.00	0.00	1.17	0.27	0.74	0.11
Dihydroxyacetone phosphate	0.71	0.18	1	0	0.35	0.15	0.44	0.24	0.68	0.23	1.00	0.39
D-pantothenic acid	1.13	0.26	1	0	1.93	0.29	2.72	1.42	1.09	0.21	1.25	0.48
D-Ribose 5-phosphate	2.12	0.62	1	0	0.14	0.04	0.11	0.04	1.44	0.38	0.75	0.11
D-Xylulose-5-phosphate	1.65	0.62	1	0	0.25	0.08	0.49	0.17	1.30	0.39	0.71	0.15
Flavin adenine dinucleotide	1.30	0.17	1	0	1.40	0.39	0.96	0.16	1.20	0.23	1.06	0.15
Guanosine	0.87	0.12	1	0	0.65	0.07	0.60	0.37	0.77	0.16	0.65	0.14
Hypoxanthine	0.94	0.09	1	0	0.78	0.09	0.79	0.28	0.98	0.21	1.04	0.20
Inosine	0.81	0.09	1	0	0.45	0.05	0.70	0.24	0.81	0.18	1.00	0.16
Inosine 5-diphosphate	2.11	0.25	1	0	0.49	0.02	1.24	0.20	1.33	0.33	0.96	0.11
Inosine 5-monophosphate	1.76	0.21	1	0	13.21	4.49	8.38	1.82	2.00	0.27	1.21	0.14
Inosine 5-triphosphate	1.72	0.39	1	0	0.06	0.03	0.07	0.04	0.68	0.18	0.58	0.08
Ketoisovaleric acid	1.06	0.25	1	0	0.93	0.06	0.95	0.13	1.00	0.30	0.82	0.17
Lactic acid	0.56	0.04	1	0	0.04	0.01	0.08	0.00	0.51	0.09	0.72	0.13
L-Aspartic Acid	1.47	0.31	1	0	1.33	0.24	1.20	0.13	1.37	0.22	1.22	0.43
L-Carnitine	2.04	0.66	1	0	2.96	0.69	3.06	0.86	1.84	0.57	1.10	0.26
L-Dihydroorotic acid	1.24	0.13	1	0	1.75	0.34	1.02	0.44	1.37	0.34	0.81	0.19
L-Glutamic acid	1.49	0.26	1	0	1.52	0.25	1.03	0.18	1.30	0.18	1.02	0.14
L-Gluthathione (oxidized)	1.42	0.26	1	0	1.34	0.43	1.74	0.48	1.29	0.29	1.02	0.16
L-Hydroxyglutaric acid	1.00	0.11	1	0	0.45	0.04	0.48	0.05	0.78	0.12	0.70	0.13
L-Kynurenine	1.27	0.17	1	0	1.83	0.50	1.20	0.31	0.96	0.26	0.79	0.14
L-Malic acid	0.70	0.04	1	0	0.70	0.08	0.80	0.05	0.74	0.02	0.82	0.19
L-Methionine	1.08	0.33	1	0	0.76	0.16	0.64	0.16	0.88	0.20	0.93	0.13
L-Phenylalanine	0.84	0.09	1	0	0.66	0.06	0.74	0.14	0.73	0.12	0.97	0.09
L-Tyrosine	1.29	0.38	1	0	0.86	0.14	0.77	0.18	0.83	0.14	0.99	0.14
N-carbamoyl-DL-aspartic acid	1.19	0.13	1	0	1.01	0.29	0.26	0.06	1.07	0.25	0.80	0.17
Orotic acid	1.06	0.07	1	0	1.33	0.29	0.65	0.10	0.72	0.14	0.62	0.08
Phenylpyruvic acid	0.99	0.26	1	0	1.46	0.75	0.60	0.13	0.62	0.24	1.04	0.56
Phosphoenolpyruvic acid	1.18	0.41	1	0	0.06	0.02	0.02	0.00	1.16	0.49	0.77	0.14
Pyruvic acid	1.30	0.14	1	0	0.35	0.07	0.45	0.19	1.12	0.26	0.90	0.16
Succinic acid	0.87	0.02	1	0	0.94	0.09	0.94	0.04	0.94	0.07	0.92	0.13
Taurine	5.14	3.52	1	0	3.48	1.59	1.95	0.66	4.62	3.22	1.14	0.32
Uracil	0.86	0.20	1	0	0.81	0.25	0.89	0.21	1.19	0.19	0.73	0.20
Uric acid	1.00	0.26	1	0	0.82	0.18	0.65	0.22	0.72	0.18	0.77	0.05
Uridine	0.97	0.12	1	0	0.83	0.07	0.91	0.34	0.89	0.18	0.85	0.15
Uridine 5-diphosphoglucose	3.93	2.02	1	0	1.51	0.73	1.06	0.70	0.68	0.19	1.22	0.61
Xanthine	0.93	0.10	1	0	0.82	0.06	0.87	0.30	0.97	0.24	0.85	0.15
Xanthosine	0.67	0.13	1	0	0.66	0.19	0.64	0.30	0.57	0.15	0.71	0.12

**FIGURE 6:**
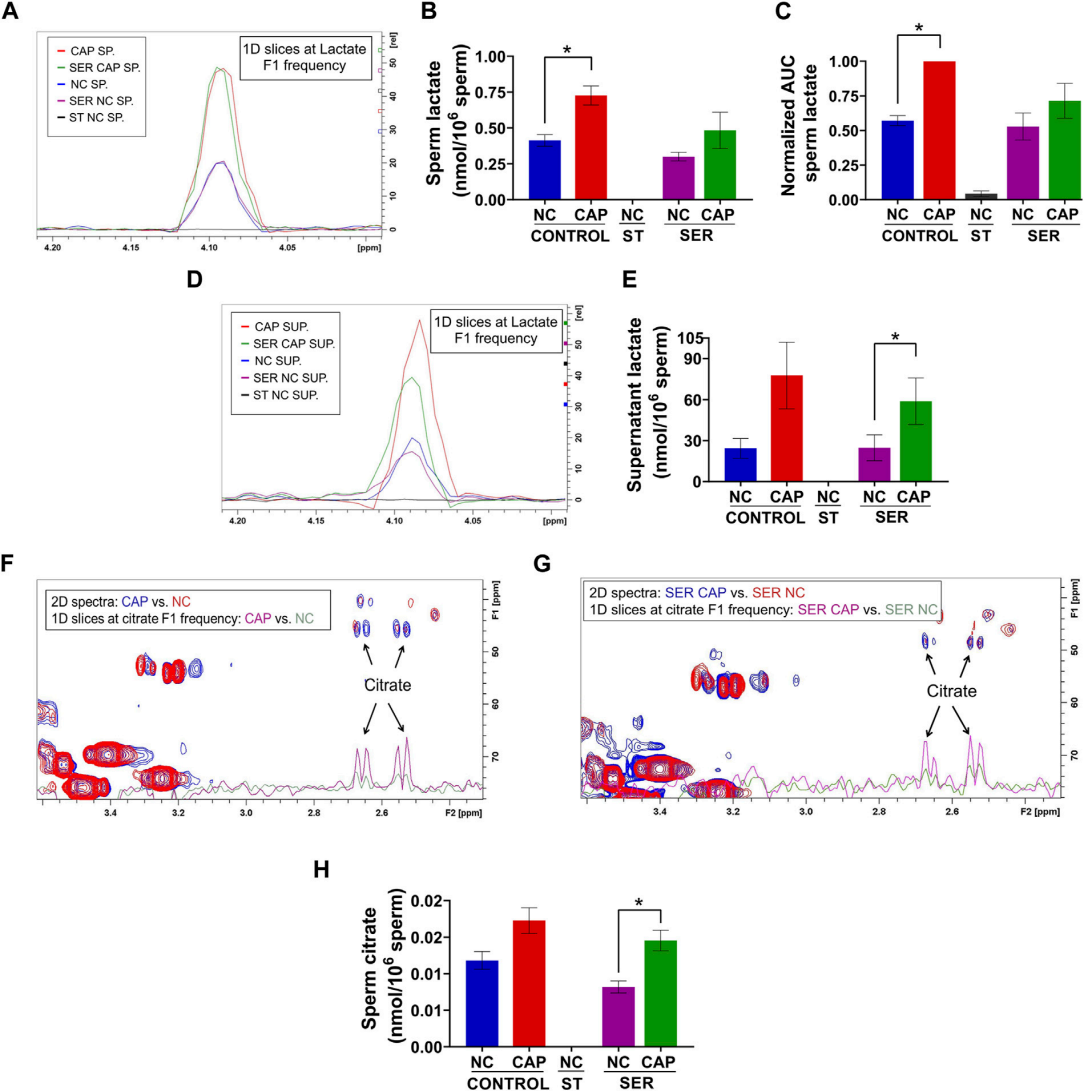
Capacitated sperm upregulate energy production metabolic pathways. Measurement of metabolites by NMR and MS was performed after incubation under the following conditions: continuously incubated with glucose in NC (without BSA and without HCO_3_^−^) or CAP (with BSA and HCO_3_^−^); continuously incubated without glucose in NC (ST); incubated without glucose in NC condition until motility stopped (∼30–40 min) and recovered with glucose in non-capacitated medium (SER-NC) or in capacitated medium (SER-CAP). **(A)** Following ^13^C-labeled glucose metabolites, 1D slices at a lactate F1 frequency of a representative 2D NMR ^1^H-^13^C HSQC experiment of sperm (SP) incubated for 60 min under the different conditions. **(B)** Sperm lactate amount determined by 2D NMR ^1^H-^13^C HSQC experiments. The results are expressed as the mean ± SEM of three independent experiments. Lactate was undetectable in NC ST sperm. T-tests between NC and CAP (control and SER) conditions were performed. * indicates a significant difference with *p* < 0.05. **(C)** AUC of the lactate MS peak of sperm incubated under the different conditions, normalized to CONTROL CAP. The results are expressed as the mean ± SEM of seven independent experiments. T-tests with Wilcoxon’s matched-pair signed rank test between NC and CAP (control and SER) conditions were performed. * indicates significant difference with *p* < 0.05. **(D–E)** Idem A-B but for the supernatant (SUP) of sperm incubated for 60 min in the different conditions. * indicates a significant difference with *p* < 0.05. **(F–G)** Following ^13^C-labeled glucose metabolites, the representative 2D NMR ^1^H-^13^C HSQC experiment showing citrate together with the 1D slices at citrate F1 frequency of the 2D spectrum of sperm incubated for 60 min under the different conditions. **(H)** Sperm citrate amount determined by 2D NMR ^1^H-^13^C HSQC experiments. The results are expressed as the mean ± SEM of three independent experiments. Citrate was undetectable in NC ST sperm. T-tests between NC and CAP (control and SER) conditions were performed. * indicates a significant difference with *p* < 0.05.

As predicted from extracellular flux analyzer experiments (Figure 3C) and our previously work (Balbach et al., 2020b; Hidalgo et al., 2020), both NMR and MS analyses confirm that lactate production is increased during capacitation in both CAP and SER-CAP (Figures 6A–C). Here, we also show that most of the lactate produced during glycolysis is extruded into the media (Figures 6D, E). Unexpectedly, we identified lactate in the starved sperm and supernatant via one-dimensional (1D) ^1^H-NMR that was not derived from ^13^C-labeled glucose (Supplementary Figures S3A, B), as it was 0 via two-dimensional (2D) ^1^H-^13^C HSQC NMR spectra (Figures 6A–B, D–E). Similarly, we observed ^13^C-acetate derived from ^13^C-glucose (Supplementary Figures S4A, B), indicating that sperm can metabolize the conversion of pyruvate to acetate, as in other cell types with high glucose consumption (Liu et al., 2018). As observed with lactate, 1D ^1^H-NMR indicates that this metabolite is also produced under starving conditions (Supplementary Figures S4C, D). Altogether, these results suggest that sperm can form lactate and acetate from glycolysis, as well as using endogenous sources. This possibility needs to be explored in future studies. Among Krebs cycle metabolites, we were only able to identify citrate by NMR and MS. Importantly, in NMR ^1^H-^13^C 2D HSQC spectra, citrate is labeled with ^13^C, which means it is derived from exogenously supplied ^13^C-glucose (Figures 6F–H). These data confirm the observations from extracellular flux analysis [(Figure 4) and (Balbach et al., 2020b)] that in sperm, as in somatic cells, glycolytic products can feed the Krebs cycle and OxPhos.

**FIGURE 7:**
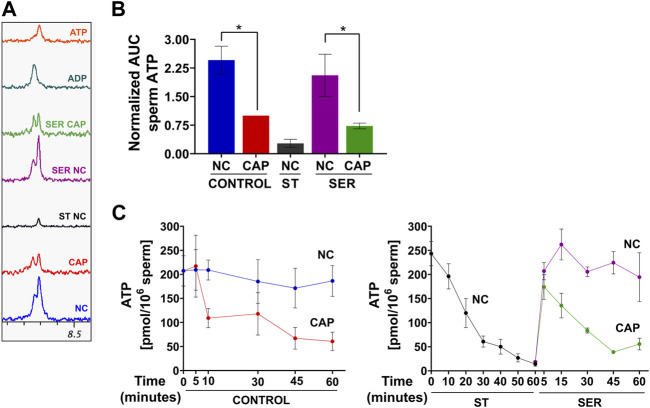
Sperm capacitation decreases intracellular ATP content. Measurement of metabolites by NMR, MS, and chemiluminescence was performed after incubation under the following conditions: continuously incubated with glucose in NC (without BSA and without HCO_3_^−^) or CAP (with BSA and HCO_3_^−^); continuously incubated without glucose in NC (ST); incubated without glucose in NC condition until motility stopped (∼30–40 min) and recovered with glucose in non-capacitated medium (SER-NC) or in capacitated medium (SER-CAP). **(A)** Representative 1D ^1^H NMR spectra showing ATP and ADP peaks of sperm incubated for 60 min under the different conditions. **(B)** AUC of the ATP MS peak of sperm incubated under the different conditions, normalized to CONTROL CAP. The results are expressed as the mean ± SEM of seven independent experiments. T-tests with Wilcoxon’s matched-pair signed rank test between NC and CAP (control and SER) conditions were performed. * indicates a significant difference with *p* < 0.05. **(C)** Intracellular sperm ATP amount measured using the ATP/ADP-Glo™ Assay kit under the different conditions. The results are expressed as the mean ± SEM of at least four independent experiments.

As expected, ATP levels were significantly reduced under starvation conditions when measured by NMR (Figure 7A), MS (Figure 7B), or chemiluminescence (Figure 7C). The reduced ATP in starved mouse sperm is a likely reason why they become quiescent; they have insufficient ATP to maintain dynein ATPase function throughout the axoneme. Unexpectedly, ATP levels are also reduced in sperm incubated in capacitating media. Metabolic profiling provides an indication of steady-state levels of metabolites, which can change due to alterations in rates of production and/or consumption. Thus, the decrease in ATP levels, despite the observed capacitation-induced increase in glycolytic activity (ECAR), OxPhos activity (OCR), and lactate production, suggests that higher ATP consumption during capacitation decreases ATP levels, alleviating the feedback inhibition of glycolytic enzymes. Consistently, the new, lower steady-state level of ATP would support the essential increased ATP production. In capacitating SER sperm during recovery, when glucose is added to starved sperm, ATP levels increase for the first 5 min, and then, in cap, but not in non-cap conditions, they decrease again (Figure 7C, right panel), indicating the time at which energy consumption pathways begin to overtake the capacitation-induced increase in ATP production via glycolysis and OxPhos.

**FIGURE 8:**
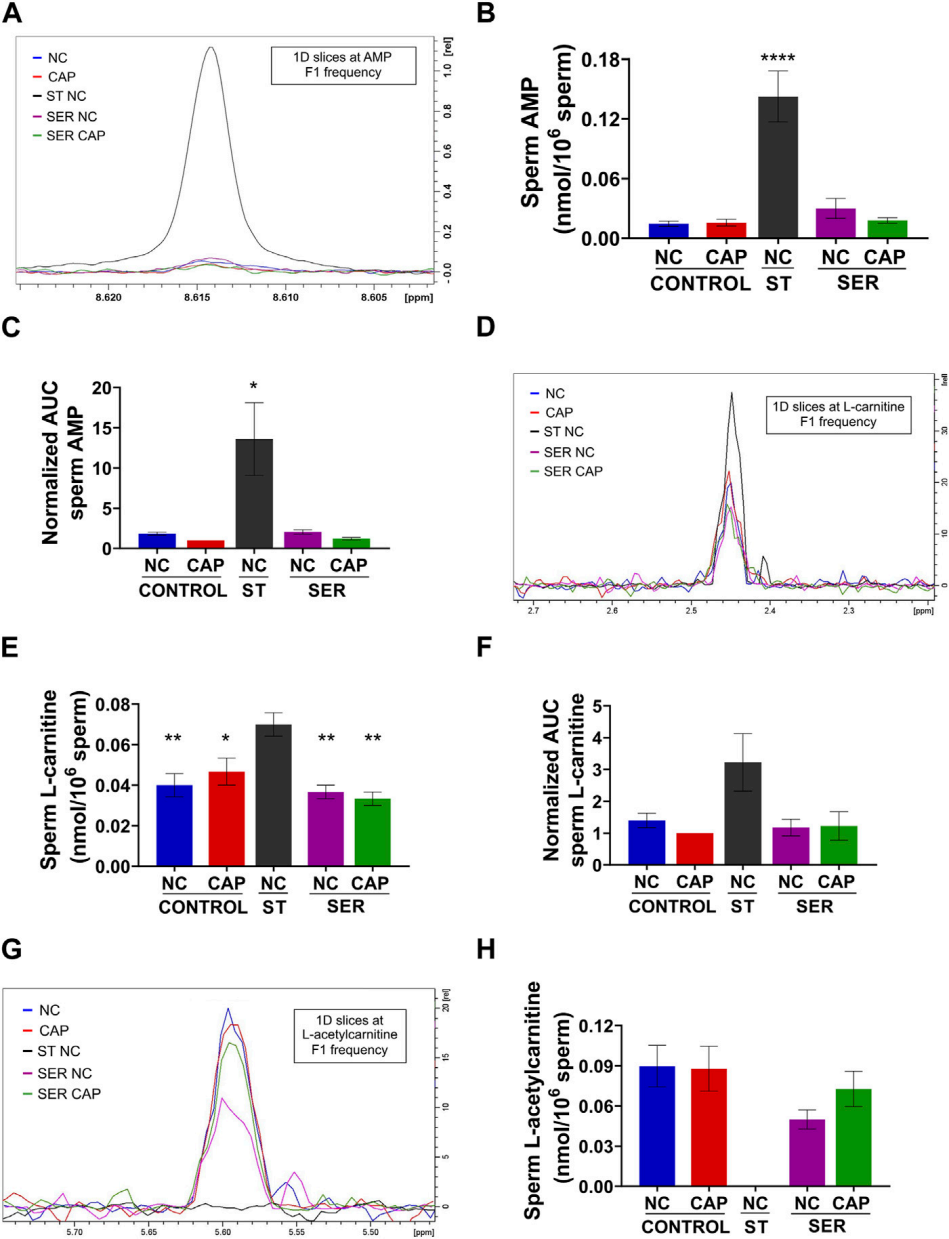
Metabolites that are significantly changed at the starving step. Measurement of metabolites by NMR and MS was performed after incubation under the following conditions: continuously incubated with glucose in NC (without BSA and without HCO_3_^−^) or CAP (with BSA and HCO_3_^−^); continuously incubated without glucose in NC (ST); incubated without glucose in NC condition until motility stopped (∼30–40 min) and recovered with glucose in non-capacitated medium (SER-NC) or in capacitated medium (SER-CAP). **(A)** Representative 1D ^1^H NMR spectra showing AMP peaks of sperm incubated for 60 min under the different conditions. **(B)** Sperm AMP amount determined by 1D NMR ^1^H experiments. The results are expressed as the mean ± SEM of four independent experiments. ANOVA with Dunnett’s multiple comparison test, comparing every condition with NC ST, was performed. **** indicates a significant difference with *p* < 0.0001. **(C)** AUC of the AMP MS peak of sperm incubated under the different conditions, normalized to CONTROL CAP. The results are expressed as the mean ± SEM of seven independent experiments. T-tests with Wilcoxon’s matched-pairs signed rank test between every condition and NC ST condition were performed. * indicates a significant difference with *p* < 0.05. **(D)** 1D slices at L-carnitine F1 frequency of a representative 2D NMR ^1^H-^13^C HSQC experiment of sperm incubated for 60 min under the different conditions. **(E)** Sperm L-carnitine amount determined by 2D NMR ^1^H-^13^C HSQC experiments. The results are expressed as the mean ± SEM of three independent experiments. ANOVA with Dunnett’s multiple comparisons test, comparing every condition with NC ST, was performed. * indicates a significant difference with *p* < 0.05; ** indicates a significant difference with *p* < 0.01. **(F)** AUC of the L-carnitine MS peak of sperm incubated under the different conditions, normalized to CONTROL CAP. The results are expressed as the mean ± SEM of four independent experiments. T-tests with Wilcoxon’s matched-pairs signed rank test between every condition and NC ST condition were performed. **(G–H)** Idem D-E but for L-acetyl carnitine. The results are expressed as the mean ± SEM of three independent experiments. L-acetyl carnitine was undetectable in NC ST sperm. T-tests between NC and CAP (control and SER) conditions were performed.

While most metabolites either decreased or remained stable during starvation (Tables 1, 2; Supplementary Figure S5), two metabolites, AMP and carnitine, were significantly increased (Figures 8A–F). AMP levels were approximately 10-fold higher in ST sperm relative to control and SER sperm as quantitated by 1D ^1^H-NMR (Figure 8B). In concert with their increased carnitine levels (Figures 8D–F), ST sperm had decreased levels of acetyl carnitine compared to control and SER sperm (Figures 8G, H). These two metabolites comprise the carnitine shuttle involved in lipid oxidation pathways, and our observation that their levels change during starvation suggests that, in the absence of external nutrients, sperm use lipid oxidation to get energy from endogenous sources.

## Discussion

When mouse sperm are incubated in the absence of external nutrients (starvation step), they stop moving but remain viable. During the rescue step, the addition of energy substrates in media which support capacitation, sperm exhibit enhanced hyperactivation, fertilization, and embryo development rates (Navarrete et al., 2019; Tourzani et al., 2022). Moreover, when transferred into pseudo-pregnant females, blastocysts derived from SER-treated sperm resulted in more pups than blastocysts derived from control sperm (Navarrete et al., 2019). As a first step to understand the molecular mechanisms underlying the benefits of SER treatment, we analyzed how this treatment affected signaling and metabolic pathways involved in sperm capacitation. Capacitation is initiated when sperm are exposed to HCO_3_^−^, which stimulates sAC to produce cAMP and consequent activation of PKA. Activation of PKA results in an FERT tyrosine kinase-dependent increase in tyrosine phosphorylation (Alvau et al., 2016). Both pathways can be monitored by Western blotting using anti-phospho-PKA substrates and anti-pY antibodies, respectively (Krapf et al., 2010). Sperm under ST-CAP conditions exhibited a transient increase in PKA-dependent phosphorylation, followed by a sustained period of dephosphorylation extending through the time when sperm stop moving (Figure 1B). These results indicate that Ser/Thr phosphatases remain active under starvation conditions. During rescue, phosphorylation pathways recovered upon re-addition of energy metabolites, and their kinetics were indistinguishable from those of controls (Figures 1C–E).

We next sought to identify changes in the metabolic profile in sperm during starvation and recovery. Our previous studies defining SER (Navarrete et al., 2015; 2016) were performed in TYH media containing glucose and pyruvate as energy substrates. We confirmed that glucose alone is sufficient to support enhanced capacitation and fertilization in SER (Figure 2) in CD1 mice, which permitted the use of media containing only glucose-derived metabolites in the absence of external pyruvate to explore the metabolic consequences of individual nutrients. Importantly, these experiments were designed to validate the use of glucose alone for metabolomics purposes, not to explore the best capacitation media for which the addition of pyruvate might be positive as was shown for human sperm (Hereng et al., 2011). Glucose can be metabolized via glycolysis and/or the pentose phosphate pathway (PPP), both cases leading to the generation of two molecules of ATP and pyruvate. In both SER and control sperm, we showed that the pyruvate formed from exogenously metabolized glucose can enter the Krebs cycle in the mitochondrial mid-piece. This conclusion is consistent with our NMR data indicating the formation of ^13^C citrate from ^13^C glucose (Figures 6F–H) (see discussion of alternatives given as follows).

We used both NMR and MS metabolomics approaches to evaluate how sperm metabolite levels change during the different treatments. Each technique has specific strengths and weaknesses, and when combined, they provide synergic and complementary knowledge. NMR spectra reveal molecules present in sperm extracts without the need for prior purification. Moreover, the same sample can be used to obtain different types of spectra, including two-dimensional ^1^H-^1^H and ^1^H-^13^C. We used NMR to follow the fate of ^13^C-glucose. However, NMR has low sensitivity, and the identification relies on comparison with available databases. In contrast, MS, which requires purification of samples under different ionization protocols for different types of molecules, provides unmatched sensitivity with more reliable metabolite identification.

In starved sperm, glycolytic intermediates were significantly reduced, and they recovered upon the addition of glucose (Table 2; Supplementary Figure S5). As predicted from extracellular flux analysis, which shows that metabolism is stimulated during capacitation (Figures 3B, C, Figure 4; Supplementary Figure S1), the metabolite profile of sperm depends on their state of capacitation (Supplementary Figure S2). The difference between non-capacitating and capacitating sperm was observed in sperm whether they were SER-treated or when they remain in the continuous presence of glucose (Supplementary Figure S2). We have shown that during capacitation sperm consume increased amounts of glucose [(Hidalgo et al., 2020) and Figure 3A]. Glucose consumed during capacitation is converted into lactate (Figures 6A–E), which is mostly secreted out of the cell (Figures 6D, E). The conversion of pyruvate, the product of glycolysis, into lactate is catalyzed by lactate dehydrogenases, including the sperm-specific lactate dehydrogenase, LDHC4. This conversion is coupled to the production of NADH oxidation to NAD^+^, a metabolite essential to maintain glycolysis at the level of the sperm-specific glyceraldehyde-3-phosphate dehydrogenase (GAPDHS) (Odet et al., 2011). Consistently, LDHC4 knockout genetic mouse models are sterile (Odet et al., 2008).

In addition to lactate secretion, we also found ^13^C acetate in the sperm incubation supernatant. This observation is consistent with that of previous work in ram (Scott et al., 1967) and bull (Melrose and Terner, 1953; Mann and Lutwak-Mann, 1981), indicating that ejaculated sperm from these species secretes ^14^C acetate when incubated in the presence of ^14^C glucose. Two independent pathways can explain pyruvate decarboxylation to form acetate. In one of them, radical oxygen species catalyze the oxidative decarboxylation of pyruvate to form acetate and CO_2_. This conversion involves the nucleophilic attack of pyruvate by H_2_O_2_ and has been shown to occur in culture mammalian cells following the fate of ^18^O-labeled H_2_O_2_. The second mechanism involved ketoacid dehydrogenases such as pyruvate dehydrogenase or alpha ketoglutarate dehydrogenase in a thiamine-dependent manner (Liu et al. (2018) to provide a detailed explanation of these molecular pathways of pyruvate decarboxylation).

In sperm, glucose is also metabolized via the PPP (Urner and Sakkas, 1999), and we detected two metabolites, xylulose-5-phosphate and ribose-5-phosphate (Table 2), which were reduced under starving conditions and restored after the addition of glucose. The PPP provides cells with NADPH. In most cells, the reducing power of NADPH is required to maintain biosynthetic anabolic reactions, but because sperm are terminally differentiated, anabolic reactions are not considered to play relevant roles during capacitation. Instead, NADPH is thought to be needed to protect sperm DNA, lipids, and other molecules from oxidative damage. While it remains unclear in which sperm compartment the PPP is active, hexokinase type I, which is the first step in both glycolysis and the PPP, is present in both the mid-piece (i.e., along with the mitochondria) and the principal piece (Visconti et al., 1996).

Extracellular flux analysis suggested that OxPhos is stimulated during capacitation (Figure 4; Supplementary Figure S1), and metabolite profiling confirmed that more exogenous glucose is metabolized into citrate in capacitated sperm relative to non-capacitated sperm (Figures 6F–H). Stimulation of OxPhos may also be relevant for sperm utilizing endogenous energy sources. Recently, we demonstrated that human sperm remain motile in the absence of external metabolites (Marín-Briggiler et al., 2021), which predicts that sperm can use endogenous energy sources such as lipids or amino acids, which is in agreement with previous reports in other sperm species (for review see Mann and Lutwak-Mann book, 1981). Although mouse sperm incubated in starvation media stopped moving in ∼ 30 min, we found that starved mouse sperm showed increased levels of carnitine with a corresponding decrease in acetyl-carnitine (Figures 8D–H). These data suggest that starved mouse sperm stimulate lipid oxidation pathways, consistent with the hypothesis that these cells can also utilize endogenous nutrients.

Not surprisingly, another metabolite that is increased in starved sperm is AMP (Figures 8A–C). Because AMP can regulate multiple aspects of metabolism, it could contribute to SER metabolic effects. AMP binds to and activates AMP-activated kinase (AMPK), which is present in mouse sperm [Supplementary Figure S6 and (Vadnais et al., 2014)] and other mammalian sperm (Tartarin et al., 2012; Martin-Hidalgo et al., 2018), senses a low energy state, stimulates glucose uptake and lipid oxidation, and inhibits anabolic reactions (Herzig and Shaw, 2018). AMP can also regulate phosphofructokinase (PFK), the rate-limiting step of glycolysis (Kamp et al., 2007). Interestingly, PFK is regulated by the ATP: AMP ratio inside cells; PFK is stimulated when AMP is high or ATP is low. While the AMP level increases during starvation, ATP levels decrease during capacitation (Figure 7C). Thus, the ATP: AMP ratio remains favorable for high PFK activity to sustain high glycolytic rates.

Although glycolysis and OxPhos are believed to occur in different compartments, we found ^13^C-citrate produced from ^13^C-glucose (Figures 6F–H). These data indicate that, at least a fraction of the pyruvate formed can render the metabolites in the Krebs cycle. How pyruvate arrives in the mitochondria is not known. Some alternatives are 1) that pyruvate diffuses from the principal piece of the mitochondria where glycolysis occurs to the mitochondria in the mid-piece; 2) that lactate derived from pyruvate is the molecule diffusing from the principal piece to the mid-piece; 3) considering that lactate and acetate are released into the incubation media (Figures 6D, E; Supplementary Figure S4B), we cannot discard the fact that once secreted, these molecules can reenter the sperm by transporters localized in the mid-piece; 4) although glycolytic enzymes have been shown to be in the principal piece (Nakamura et al., 2008; Nakamura et al., 2010), it is not possible to discard the fact that lower levels of these enzymes are present in the mid-piece. The efficiency of this coupling warrants further studies. Coupling between glycolysis and oxidative phosphorylation has been reported for sperm of other species (Mann and Lutwak-Mann, 1981), and it is suggested by our previous and current findings that glucose alone is sufficient for the capacitation-induced increase in OCR Seahorse parameters; 5) despite this evidence, as a fifth alternative, we cannot discard the fact that the glucose-derived citrate formed as a result of a cytosolic reaction since cytosolic citrate synthase has been shown to be present in mammalian sperm outside the mitochondrial compartment (Kang et al., 2022).

In summary, in this paper, we applied methods for studying metabolism in sperm during capacitation as well as following starvation and recovery. Following the fate of ^13^C-glucose by NMR allowed quantitative assessment of glycolysis and provided direct evidence that large amounts of glucose-derived pyruvate are released into the media after conversion to lactate and acetate (Figures 6D, E; Supplementary Figure S4B). In addition, our results indicate a significant increase in AMP (Figures 8A–C), which is likely the consequence of ATP hydrolysis. In addition to the aforementioned possible roles of AMP and carnitine, reduced levels of ATP are expected to affect multiple signaling pathways relevant for sperm capacitation, such as phosphorylation and ion homeostasis. For example, we recently showed that when sperm incubated under starving conditions become immotile, intracellular Ca^2+^ ([Ca^2+^]_i_) levels are highly increased (Sánchez-Cárdenas et al., 2021). This increase in [Ca^2+^]_i_ is likely due to inactivation of the ATP-dependent Ca^2+^ ATPase PMCA4. The starvation-induced increase in [Ca^2+^]_i_ also occurs in sperm lacking the sperm-specific Ca^2+^ channel complex CatSper (Sánchez-Cárdenas et al., 2021), suggesting that, in addition to the CatSper complex, other Ca^2+^ transporters are present in mouse sperm. It is noteworthy that SER treatment rescued the infertile phenotype of sperm *in vitro* from mice deficient in C2CD6, one of the CatSper subunits (Yang et al., 2022). In bovine sperm, an increase in [Ca^2+^]_i_ under ATP starvation by pharmacological inhibition of glycolysis and OxPhos was reported, but the calcium influx was reported to be via CatSper (Dahan and Breitbart, 2022). Finally, one of the most surprising consequences of starvation and recovery is the effects on post-fertilization events such as embryo development (Navarrete et al., 2019; Tourzani et al., 2022; Al-Hafedh and Cedden, 2023). We speculate that SER improvements in embryo development rates are due to changes in the sperm epigenetic information. In somatic cells, it is well-established that histone-modifying enzymes are regulated by changes in metabolites (Reid et al., 2017). Although the sperm chromatin is highly condensed due to histone replacement by protamine during spermiogenesis, a considerable number of histones remain in mature sperm (Jung et al., 2017). Then, the metabolite changes observed during SER treatment could impact posttranslational modifications of sperm-retained histones and affect early embryo development after fertilization. Alternatively, reduced levels of ATP can affect the concentration of small non-coding RNAs in sperm, which have also been proposed to mediate epigenetic transmission to the embryo (Sharma et al., 2016; Conine et al., 2018). Although, at the moment, it is too early to understand how the SER sperm regulates early embryo development, studies such as ours are a necessary step to gain an insight into why SER-treated sperm exhibit increased sperm functionality.

## Material and methods

### Reagents

The following chemicals were purchased from the given sources (codes between parentheses indicate the catalog number of the respective compound): sodium bicarbonate (NaHCO_3_) (S-5761); bovine serum albumin (BSA) (fatty acid-free) (A0281), Tween-20 (P7949), fish skin gelatin (G-7765), concanavalin A (L7647), dbcAMP (D0627), IBMX (I5879), and rabbit monoclonal anti-phospho-PKA substrates (anti-pPKAS) (7,906) were purchased from Sigma (St. Louis, MO, United States). Anti-phosphotyrosine (anti-pY) monoclonal antibody (clone 4G10) was obtained from Millipore (Billerica, MA, United States). Horseradish peroxidase-conjugated anti-mouse and anti-rabbit IgGs were purchased from Jackson ImmunoResearch Laboratories (West Grove, PA, United States) and GE Life Sciences (Pittsburgh, PA, United States), respectively. Acrylamide/bis solution (30%) (161-0138) and β-mercaptoethanol (BP176-100) were obtained from Bio-Rad (Hercules, CA, United States). HEPES (BP310-100) was purchased from Roche (Hatfield, PA, United States), and the Amplex®Red Glucose/Glucose Oxidase Assay Kit was obtained from Invitrogen (Grand Island, NY, United States) (A22189). D-glucose (U-13C6, 99%) (CLM-1396), TMSP-2,2,3,3-D4 (D, 98%), sodium-3-trimethylsilylpropionate (DLM-48), maleic acid disodium salt monohydrate (13C4, 99%) (CLM-10892), deuterium oxide (D, 99.9%) (DLM-4-100), and HEPES (D18, 98%) (DLM-3786) were purchased from Cambridge Isotope Laboratories (Tewksbury, MA, United States). ADP/ATP-GloTM was obtained from Promega (Madison, WI, United States), and Seahorse XFe96 FluxPak mini was purchased from Agilent (102601-100) (Santa Clara, CA, United States).

### Animals

All procedures involving experimental animals were performed in accordance with Protocol 2019–0008 approved by the University of Massachusetts Amherst Institutional Animal Care and Use Committee (IACUC). Cauda epididymal mouse sperm were collected from CD1 retired male breeders (Charles River Laboratories, Wilmington, MA, United States). Mouse oocytes were collected from 8–10-week-old superovulated CD1 females (Charles River Laboratories, Wilmington, MA, United States). For superovulation, females were injected with 5 IU pregnant mare serum gonadotropin (PMSG) (Lee BioSolutions, cat #493-10) and 5 IU human chorionic gonadotrophin (hCG) (Sigma, cat #CG5) 48 h later, and cumulus–oocyte complexes (COCs) were collected 13 h post-hCG injection.

### Media

Modified Toyoda–Yokoyama–Hosi (mTYH) medium was used for sperm (Toyoda et al., 1971). The non-capacitating (NC) medium used contained the following: NaCl (119.3 mM), KCl (4.7 mM), CaCl_2_.2H_2_O (1.71 mM), KH_2_PO_4_ (1.2 mM), MgSO_4_.7H_2_O (1.2 mM), HEPES (20 mM), glucose (5.56 mM), and sodium pyruvate (0.51 mM). For capacitating (CAP) conditions, 15 mM NaHCO_3_ and 5 mg/mL BSA were added. These media were also used containing only glucose (5.56 mM) or only pyruvate (0.51 mM) or devoid of both energy substrates (starving (ST) medium). In all cases, pH was adjusted to 7.2–7.4 with NaOH. For glucose consumption measurements, glucose was used at a final concentration of 1 mM (Hidalgo et al., 2020). For NMR experiments, ^13^C uniformly labeled glucose (5.56 mM) and deuterated HEPES (20 mM) were used (NMR TYH). For Seahorse experiments, HEPES was used at a final concentration of 1 mM (Seahorse TYH). Both non-capacitating and capacitating media contained 5 mg/mL BSA. The capacitating medium also contained dbcAMP (1 mM) and IBMX (0.1 mM), as shown previously (Balbach et al., 2020a).

The Toyoda–Yokoyama–Hosi (TYH) medium (IVF TYH) was used for sperm fertilization assay, consisting of NaCl (119.3 mM), KCl (4.7 mM), CaCl_2_.2H_2_O (1.71 mM), KH_2_PO_4_ (1.2 mM), MgSO_4_.7H_2_O (1.2 mM), glucose (5.56 mM), sodium pyruvate (0.51 mM), NaHCO_3_^−^ (25.1 mM), 4 mg/mL BSA, 10 μg/mL gentamicin, and 0.0006% phenol red at pH 7.4 equilibrated with 5% CO_2_. This medium was also used containing only glucose (5.56 mM) or only pyruvate (0.51 mM) or devoid of both energy substrates (ST medium). For oocyte collection, Tyrodes’s lactate–HEPES (TL-HEPES) was used, consisting of NaCl (114 mM), KCl (3.22 mM), CaCl_2_.2H_2_O (2.04 mM), NaH_2_PO_4_.2H_2_O (0.35 mM), MgCl_2_.6H_2_O (0.49 mM), NaHCO_3_^−^ (2.02 mM), lactic acid (sodium salt) (10 mM), and HEPES (10.1 mM) at pH 7.4.

### Sperm incubation and SER treatment

Male mice were culled, and both cauda with 3–5 excisions were placed in 2 mL ST medium. We let sperm swim out for 15 min at 37°C. Cauda were removed, and sperm were washed for 5 min at 300 × g and resuspended in 2 mL ST medium. Samples were centrifuged for an additional 5 min at 150 × g at RT, supernatants removed, and sperm resuspended in ST medium. Sperm from the different mice were pooled together, and an aliquot was separated for sperm counting. During collection and washing (∼20 min), sperm remained motile, which were then separated and incubated under six different conditions. Incubation conditions 1 (NC) and 2 (CAP): for regular NC and CAP conditions, immediately after washing, sperm were resuspended in new tubes containing media to create 1× NC and CAP conditions. After 60 min of incubation, these tubes were processed. Incubation conditions 3 (ST-NC) and 4 (ST-CAP): for starved in NC and for starved in CAP conditions, pooled sperm were resuspended in either the same ST media or in ST media containing BSA and HCO_3_^−^. Upon resuspension, sperm were continuously checked until they stopped moving (∼30–40 min). Once sperm stopped moving, they were processed to be used. Incubation conditions 5 (SER-NC) and 6 (SER-CAP): sperm were incubated in the ST medium and continuously checked until they stopped moving. Once they stopped, they were transferred to be rescued in new tubes containing media with the respective nutrient (either glucose or pyruvate or both) either under 1× NC (SER-NC) or 1x CAP (SER-CAP) conditions. These last two conditions were ready to be processed after 60 min of incubation. In all experiments, sperm concentration varied between 2 and 5 million/ml. For IVF experiments SER treatment was carried out as previously stated by Navarrete et al. (2019); Navarrete et al. (2016). Briefly, sperm were incubated in the IVF TYH ST medium. Once the sperm stopped moving, a fraction of them was resuspended in IVF TYH medium containing glucose, pyruvate, or both energy substrates and was used for insemination.

For Seahorse experiments, we adapted the procedure previously described (Balbach et al., 2020a). Briefly, after washing in the ST Seahorse TYH medium, sperm were aliquoted to tubes containing the NC medium with glucose and BSA or to tubes containing the ST medium with BSA. Then, sperm under both conditions were plated (180 µL) in Seahorse-compatible well plates, and after centrifuging, the cell cartridge was placed in the Seahorse equipment. At this point, sperm in the ST medium were already starved for ∼20 min. Because those sperm inside the Seahorse will be under starving conditions for an additional 10 min (before the addition of the medium with glucose for SER sperm or before more starving medium for ST and never rescued sperm), sperm under the SER condition will be starved for a total time of ∼30–40 min (the time that it usually takes for the sperm to stop moving).

### Western blot

Upon the completion of the aforementioned different treatments, sperm were collected by gentle centrifugation (3,000 × g), washed in 1 mL of PBS, resuspended in Laemmli sample buffer (Laemmli, 1970) without β-mercaptoethanol, boiled for 5 min, and centrifuged at 12,100 × g. The pellets were discarded and supernatants supplemented with 5% β-mercaptoethanol and boiled for 4 min. Protein extracts were separated by 8% SDS-PAGE and electro-transferred to PVDF membranes (Millipore). Immunoblotting was conducted with anti-pPKAs and anti-pY antibodies sequentially as previously described (Krapf et al., 2010). Briefly, PVDF membranes were blocked with 5% fat-free milk in TBS containing 0.1% Tween 20 (T-TBS) for anti-pPKAs and with 5% fish gelatin for anti-pY in PBS containing 0.1% Tween 20 (T-PBS). Antibodies were used at a final concentration of 1:10.000. Secondary antibodies were diluted in T-TBS or T-PBS (1:10.000) for anti-pPKAs and anti-pY, respectively. Before conducting anti-pY Western blotting, the PVDF membranes used for pPKAs were stripped at 55°C for 20 min in 2% SDS, 0.74% β-mercaptoethanol, and 62.5 mM Tris, pH 6.5, and then washed six times for 5 min each in T-TBS prior to reprobing. An enhanced chemiluminescence ECL Plus Kit (GE Healthcare) and ECL regular were used for the detection of pPKAs and pY signals, respectively. Quantitative analysis was performed using ImageJ 1.47 V software (National Institutes of Health, United States). Regions of interest (ROIs) used for quantification are indicated by a # on the left of the respective Western blot. The extent of hexokinase tyrosine phosphorylation (see arrow in Figure 1A, lower panel) does not change during capacitation (Porambo et al., 2012; Chung et al., 2014) and was used as the loading control. The optical density of the bands was measured and relativized to tyrosine-phosphorylated hexokinase.

### Computer-assisted sperm analysis measurements

Sperm suspensions (30 μL; 2 × 10^6^ sperm/ml) from the different treatments were loaded into a pre-warmed chamber slide (depth 100 µm) (Leja slide, Spectrum Technologies, Aurora, IL, United States) and placed on a microscope stage at 37°C. Sperm motility was examined using the CEROS computer-assisted sperm analysis (CASA) system (Hamilton Thorne Research, Beverly, MA, United States). The default settings include the following parameters: frames acquired: 90; frame rate: 60 Hz; minimum cell size: 4 pixels; static head size: 0.13–2.43; static head intensity: 0.10–1.52; static head elongation: 5–100. At least five microscopy fields corresponding to a minimum of 200 sperm were analyzed for each treatment in each experiment. Hyperactivated sperm were those having the following parameters: curvilinear velocity (VCL) > 271.00 μm/s, linearity (LIN) < 50.00%, and amplitude of lateral head (ALH) > 3.50 µm.

### *In vitro* fertilization

COCs were collected in TL-HEPES 13 h post hCG and washed under respective TYH medium conditions (with glucose and pyruvate, with glucose only, or with pyruvate only) prior to being placed in the insemination droplet under the proper TYH medium conditions (with glucose and pyruvate, with glucose only, or with pyruvate only). Sperm were incubated as described previously, and approximately 100,000 sperm were added to a 90 µL insemination droplet. After 4 h, oocytes were washed in TYH (containing glucose and pyruvate) and allowed to culture for 20 h. The following day, fertilization was determined by visualization of cleavage into a 2-cell stage embryo.

### Glucose consumption measurement

Glucose consumption measurement was performed as previously described by Hidalgo et al. (2020); Hidalgo et al. (2020). Briefly, sperm under the different conditions were prepared as described previously, and 50 μL of the sperm suspension was taken every hour for 3 h of incubation. Sperm were then removed by centrifugation at 12,000 × g for 3 min, and the supernatant was recovered and frozen at −80°C until analysis. The glucose concentration was measured using the fluorescent Amplex®Red Glucose/Glucose Oxidase Assay Kit. Fluorescence was measured in duplicate in 96-well plates (Corning^®^#3915, New York, United States). In each well, 3 μL of the sample was added to 47 μL of 1x reaction buffer followed by 50 μL of the reaction mix. The reaction mix contained (concentrations are given in parentheses) Amplex^®^Red reagent (100 μM), horseradish peroxidase (0.2 U/ml), and glucose oxidase (2 U/ml). After 30 min of incubation at 23°C, fluorescence was measured using the fluorimeter capabilities of the POLARstar Omega equipment (BMG LABTECH, Germany) with a wavelength excitation of 540 nm and wavelength emission of 590 nm. Each assay included a “blank” sample containing the mTYH medium without glucose and standard concentration curves obtained in the presence and in the absence of BSA. After correction of all relative light unit (RLU) values with background measurement (blank sample), RLU values were averaged for each sample, and the glucose concentration was determined using the linear equation of the glucose standard curve (y = mx + b), where “y” was RLU, “x” is the glucose concentration, “m” is the slope, and “b” is the y-intercept. To calculate glucose consumption, we subtracted the remnant glucose concentration in the media that we measured after 1, 2, and 3 h of incubation from the initial glucose concentration, equal to 1 mM (time 0). These glucose concentration data were represented over time. Each equation line was calculated, and the slope represented the average of glucose consumption by unit time (hour) over a 3-h incubation period corrected by the sperm concentration in each experiment. This value was used to calculate the average ± SEM of each independent measurement indicated as the glucose consumption rate.

### Seahorse measurements

Seahorse measurements were performed as previously described by Balbach et al. (2020a). In brief, the sensor cartridge was hydrated with H_2_O overnight in a 37°C non-CO_2_ incubator. The extracellular flux analyzer 96-well cell plate was coated with 0.5 mg/mL (w/v) concanavalin A and dried overnight at RT. The following morning, H_2_O in the utility plate was replaced with 200 μL calibrant per well and incubated for at least 1 h in a 37°C non-CO_2_ incubator. Port A was filled with Seahorse TYH containing glucose or with ST Seahorse TYH. Port B was filled with 10 mM dbcAMP and 1 mM IBMX in every even number column and 1 mM DMSO (vehicle) in every uneven column, in the corresponding medium (Seahorse TYH containing glucose or with ST Seahorse TYH). The injection of the port B content to sperm starts the recovery process and capacitation in the corresponding wells (as the addition creates dilution of 10 times, glucose, dbcAMP, and IBMX are 10 X). For one plate, sperm from six mice were isolated as described previously. After washing with the ST Seahorse TYH medium, sperm were aliquoted to tubes containing 3 mL NC Seahorse TYH medium with glucose and BSA or to tubes containing ST Seahorse TYH medium with BSA. Then, 180 µL of sperm suspension (1.2 × 10^6^ sperm/well) under both conditions was plated in the corresponding wells of the concanavalin A-coated cell plate. The four corner wells were filled with the Seahorse TYH medium only for background correction. The plate was centrifuged at 250 × g for 1 min, rotated by 180°, and centrifuged again at 250 × g for 1 min.

A template was generated in the Agilent Seahorse XFe96 analyzer according to these details: one cycle of basal measurement, port A injection and one cycle of measurement, and port B injection and 18 cycles of measurement. Each cycle of measurement was 2 min of mixing plus 3 min of measurement. For each condition, 7/8 replicates were measured. After successful calibration, the utility plate was replaced with the sperm plate. The Seahorse analyzer kinetically and simultaneously measures glycolysis and OxPhos. The conversion of glucose to lactate by glycolysis with lactate being the primary source of free protons acidifies the medium. Glycolysis is determined by measuring the rate of proton release or the extracellular acidification rate (ECAR). OxPhos is determined by measuring the oxygen consumption rate (OCR).

Data were analyzed by first removing the first data point (basal measurement) and subtracting the first time point to every other time point to make all data start at the zero time point. Then, every replicate was subtracted with its initial value to make all of them start at 0, showing the change from its initial value (Δ ECAR or Δ OCR). The mean of all the replicates was obtained, and the area under that curve (AUC) was calculated. To compare between experiments, AUC data were normalized: all AUCs were subtracted with the minor of them and then divided by the value of control CAP (for control conditions) or by the value of the SER-CAP condition (for starved and SER conditions) (normalized AUC Δ ECAR or normalized AUC Δ OCR).

### Nuclear magnetic resonance

#### NMR sample preparation

For every condition, sperm from six mice (∼90 × 10^6^ sperm/condition) were isolated and incubated as described previously. When tubes were ready to be processed, the supernatant and sperm were separated by centrifugation for 5 min at 300 × g at RT. The supernatant was transferred to a 15-mL falcon tube. A volume of 100 μL of the supernatant was transferred to a new tube, and ice-cold methanol was added to obtain a final concentration of 80% methanol. The tubes were incubated for 5 min in dry ice and then vortexed and then moved to wet ice and stored at −80°C overnight. Sperm were washed with PBS twice, and the supernatant was removed. Ice-cold methanol was added to the sperm pellet to obtain a final concentration of 80% methanol. The tubes were incubated for 5 min in dry ice and vortexed until the pellet was resuspended. The tubes were moved to wet ice and stored at −80°C overnight. As controls, the different media were also treated with 80% methanol, in the same way used for the samples. Supernatant samples, sperm samples, and control media were centrifuged for 20 min at 20,000 × g at 4°C. Supernatants were transferred to a new tube on ice, dried using a compact, integrated vacuum concentrator (SpeedVac™), and stored at −80°C until NMR analysis. Samples and controls were resuspended in 520 μL of 5 μM 3-(trimethylsilyl) propionic-(2,2,3,3-d4) acid sodium salt (TSP) and 25 μM ^13^C maleic acid solution in D_2_O. TSP was used as the frequency standard (δ = 0.00 ppm), and ^13^C maleic acid was used for quantification of ^13^C glucose metabolites in 2D ^1^H-^13^C HSQC spectra. Aliquots of 520 μL were transferred into standard 5-mm NMR tubes for NMR measurements.

#### NMR experiments and data processing

All NMR spectra were acquired on a Bruker AVANCE III solution-state NMR spectrometer equipped with a liquid helium-cooled QCI (H/F, C, N, P), deuterium lock, and a cryoprobe operating at a frequency of 600.259972 MHz for proton and 150.934614 MHz for carbon. All NMR data were collected at T = 298 K. Nonuniform sampling (NUS) schedules were generated using a Poisson gap distribution with a sinusoidal weight of 2 and a random seed generator. The same 50% NUS schedule and seed were used for all ^1^H-^13^C HSQC experiments. The spectral widths along the direct ^1^H and the indirect ^13^C dimensions were set at 6,009.615 and 19,623.264 Hz, respectively. The number of complex points in the direct dimension was set at 1 K, and in the indirect dimension, it was set at 105 with a 50% NUS sampling schedule for ^1^H-^13^C HSQC experiments. The 50% NUS schedule and seed were used for ^1^H-^1^H TOCSY experiments. The spectral width along both the direct and indirect ^1^H dimensions was set at 6,009.615 Hz. The number of complex points in the direct dimension was set at 1,024, and in the indirect dimension, it was set at 512 with a 50% NUS sampling schedule. The number of scans for the 1D ^1^H experiments was set to 256 (d1 = 10.0 s). The number of scans for the 2D ^1^H-^13^C HSQC experiments was set to 96 (d1 = 2.0 s). The transmitter frequency offset was set to 75 ppm in the ^13^C dimension and 4.7 ppm in the ^1^H dimension. The number of scans for the 2D ^1^H-^1^H TOCSY experiments was set to 16 (d1 = 2.0 s). The transmitter frequency offset was set to 4.7 ppm in both the ^1^H dimensions. The spectral data were processed using the TopSpin 3.6.4 software package. The NUS data were reconstructed using the cs mode in TopSpin 3.6.4 to generate the same number of direct dimension data points and twice the number of indirect dimension data points. Both the NUS and uniform sampling (US) NMR data were zero-filled, Fourier-transformed, and manually phase-corrected to yield a final digital resolution of 2,048 (N2) 2,048 (N1) points.

### Data analysis

We assigned the metabolite peaks by comparing the chemical shifts of the 1D ^1^H, 2D ^1^H-^13^C HSQC, and ^1^H-^1^H TOCSY NMR spectra with those from the reference spectra available in the Biological Magnetic Resonance Data Bank (BMRB) (Ulrich et al., 2008) and the Human Metabolome Database (HMDB) (Wishart et al., 2007). Quantification of metabolites was performed using Bruker TopSpin 3.6.4 software. Most metabolites (lactate, glucose, citrate, carnitine, acetyl carnitine, and acetic acid) were quantified in 2D ^1^H-^13^C HSQC spectra using 25 μM ^13^C-labeled maleic acid as an internal standard. NAD, ATP, ADP, and AMP were quantified in 1D ^1^H spectra using 5 μM TSP as an internal standard.

### Mass spectrometry

#### LC-MS sample preparation

For every condition, sperm (5 × 10^6^ sperm/tube) were isolated and incubated as described previously. Sperm conditions and control media were run in quadruplicates. Sample prep for MS was the same as for NMR. Briefly, when tubes were ready to be processed, sperm were separated by centrifugation for 5 min at 300 × g, RT and washed with PBS twice. Ice-cold methanol was added to the sperm pellet to obtain a final concentration of 80% methanol, incubated for 5 min in dry ice, and vortexed until the pellet was resuspended. The tubes were then moved to wet ice and incubated at −80°C overnight. The following day, sperm samples were centrifuged for 20 min at 20,000 × g at 4°C. Supernatants were transferred to a new tube on ice, SpeedVacced, and stored at −80°C until running MS. Samples were sent on dry ice to the Memorial Sloan Kettering Cancer Center, New York, NY, United States. For sample resuspension for LC-MS/MS analysis, the tubes were placed on wet ice, and 30 µL of mobile phase A (MPA) was added and vortexed until the pellet was resuspended. Tubes were incubated on ice for 20 min, vortexing every 5 min. Samples were centrifuged for 20 min at 20,000 × g, 4°C, and 25 µL of the supernatant was transferred into LC vials for injection.

#### LC-MS/MS analysis

Ion-pair LC-MS/MS analysis was performed by LC separation on a Zorbax RRHD Extend-C18 column (150 mm × 2.1 mm, 1.8 µm particle size, Agilent Technologies) and using a gradient of solvent A (10 mM tributylamine and 15 mM acetic acid in 97:3 water: methanol) and solvent B (10 mM tributylamine and 15 mM acetic acid in methanol) according to the manufacturer’s instructions (MassHunter Metabolomics dMRM Database and Method, Agilent Technologies).

#### Intracellular ATP measurements

For every condition, sperm were isolated and incubated as described previously. To determine nucleotide concentration, sperm were centrifuged at 1,700 × g for 5 min, and supernatants were discarded. Sperm pellets were resuspended in 80 µL of boiling lysis buffer (Tris 100 mM and EDTA 4 mM, pH 7.75), and the suspension was incubated at 95°C for 5 min to allow the release of nucleotides. Finally, cells were removed by centrifugation at 12,000 × g for 5 min, and the final volume of the remaining supernatant was corrected to 100 µL with lysis buffer and stored at −80°C until use. ATP concentration was measured using a commercial firefly luciferin–luciferase assay following the manufacturer’s instructions (ADP/ATP-Glo™). Briefly, 15 µL of each sample (sperm lysate) was added to a well of a 96-well white wall clear bottom plate (Corning^®^ # 3392, New York, United States). Then, 5 µL of 4% trichloroacetic acid solution (TCA) was added to each well and incubated for 10 min at 23°C. Then, 5 µL of the neutralization solution was added to each well and incubated for 5 min at 23°C. Then, 25 µL of the ATP detection reagent was added, and after 10 min of incubation at 23°C, bioluminescence was measured using a microplate luminometer (POLARstar Omega, BMG LABTECH, Germany) controlled by Omega software (Version 5.11, BGM LABTECH, Germany). Standard curves were generated according to the manufacturer’s instructions using ATP concentrations ranging from 0 to 200 picomoles. All samples and standards were measured in duplicate. Relative light units (RLU) obtained from the blank sample were subtracted from the RLU measured for each ATP standard concentration and samples incubated under different conditions. After correction, RLU were averaged for each sample, and the ATP concentration was determined by using the linear equation generated using the ATP standard curve (y = mx + b), where “y” was the RLU, “x” was the ATP concentration, “m” was the slope, and “b” was the y-intercept.

#### Statistical analysis

Statistical analyses were performed using GraphPad Prism 8.0.2 (GraphPad Software). All data are shown as the mean ± SEM. Statistical significance between two groups was determined using two-tailed t-tests for parametric data and the Wilcoxon matched-pair signed rank test for nonparametric data. Statistical significance between multiple groups was determined using one-way ANOVA with Tukey’s or Dunnett’s multiple comparison test for parametric data and with Friedman’s test and Dunn’s multiple comparison test for nonparametric data. The statistical test performed in each figure is indicated in the Figure legend. Differences were considered significant if ∗*p* < 0.05, ∗∗*p* < 0.01, ∗∗∗*p* < 0.001, and ∗∗∗∗*p* < 0.0001. For NMR data statistical analysis, each NMR spectrum was reduced to 250 frequency bins (0.04 ppm bin size) using Bruker AMIX software version 4.01. A bin is the sum of the intensity values for a fixed number of consecutive points. Spectral regions within the range of 0 ppm–10 ppm were analyzed after deleting the regions between 4.7 and 4.86 ppm and between 3.06 and 3.34 ppm that contained the residual water peak and the methanol signal, respectively. NMR spectra were normalized (such that the total intensity of each spectrum is equal to 1) and mean-centered prior to statistical analysis. PCA was performed using the AMIX tool-kit, suitable for performing various types of multivariate data analysis and multi-group data analysis and visualizing the results. For MS PCA, AUC values for each condition were z-scored. PCA was performed using the Python scikit-learn library.

## Data Availability

The original contributions presented in the study are included in the article/Supplementary Material, further inquiries can be directed to the corresponding authors.
