# Supplementary material for: Mouse sperm energy restriction and recovery (SER) revealed novel metabolic pathways
Source: Front Cell Dev Biol. 2023 Aug 15;11:1234221. doi: 10.3389/fcell.2023.1234221 (PMC10466171; doi:10.3389/fcell.2023.1234221)
Supplement: Supplementary file 1 [file DataSheet1.PDF]

Supplementary figure 1

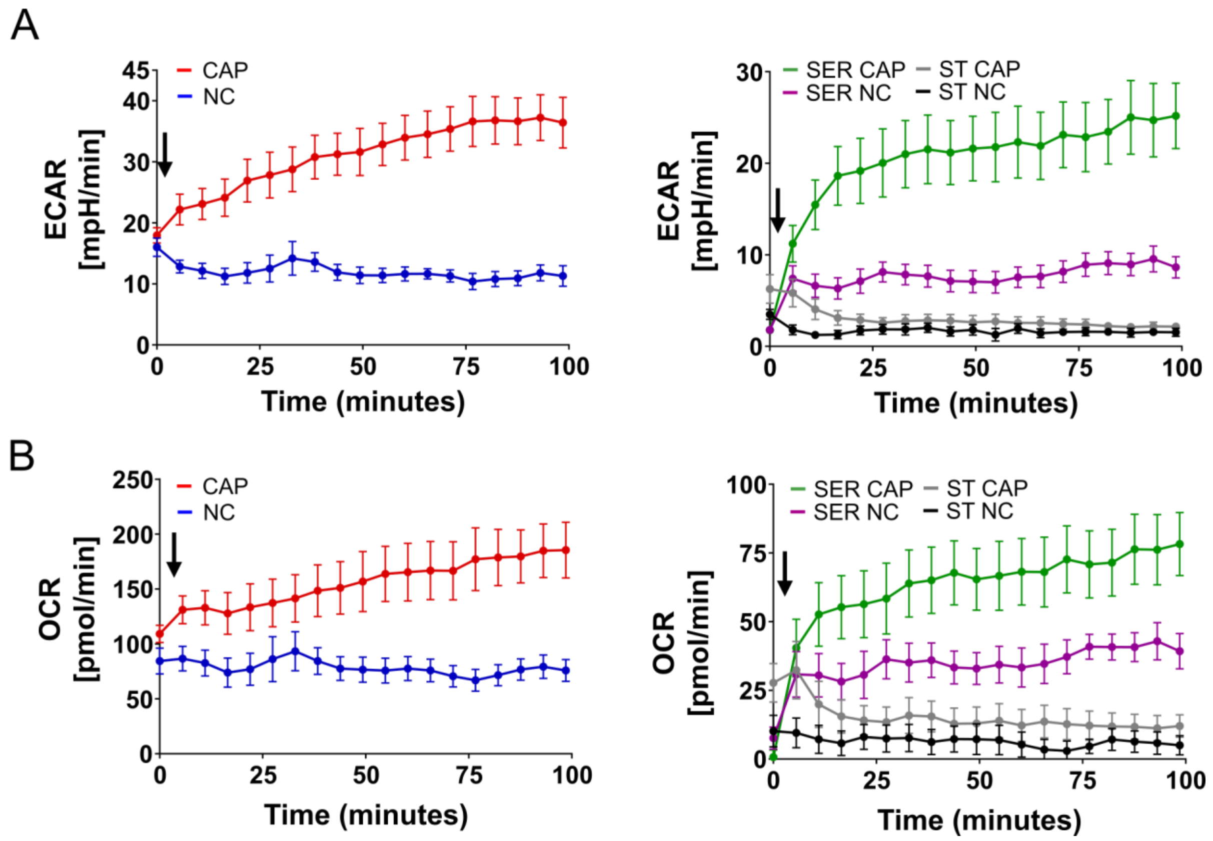

Supplementary Figure 1. Extracellular acidification rate (ECAR) and oxygen consumption rate (OCR) by Seahorse was measured under the following incubation conditions: continuously incubated with glucose in NC (without BSA and without HCO_3^-) or CAP (with dbcAMP and IBMX); continuously incubated without glucose in NC (ST-NC) or CAP (ST-CAP); incubated without glucose in NC condition until motility stopped (~ 30 to 40 min) and recovered with glucose in non-capacitated medium (SER-NC) or in capacitated medium (SER-CAP). **(A)** Representative curves of ECAR measurements in sperm incubated in the different conditions. **Left panel.** The arrow indicates the release of the content of port B (5.6 mM glucose TYH medium with DMSO (vehicle) for wells with NC sperm; 5.6 mM glucose TYH medium with 10 mM dbcAMP and 1 mM IBMX for wells with CAP sperm). **Right panel.** The arrow indicates the release of the content of port B (starving TYH medium with DMSO (vehicle) for wells with ST NC sperm; starving TYH medium with 10 mM dbcAMP and 1 mM IBMX for wells with ST CAP sperm; 56 mM glucose TYH medium with DMSO (vehicle) for wells with SER NC sperm; 56 mM glucose TYH medium with 10 mM dbcAMP and 1 mM IBMX for wells with SER CAP sperm). **(B)** Representative curves of OCR measurements in sperm incubated in the different conditions. **Left panel.** The arrow indicates the release of the content of port B (5.6 mM glucose TYH medium with DMSO (vehicle) for wells with NC sperm; 5.6 mM glucose TYH medium with 10 mM dbcAMP and 1 mM IBMX for wells with CAP sperm). **Right panel.** The arrow indicates the release of the content of port B (starving TYH medium with DMSO (vehicle) for wells with ST NC sperm; starving TYH medium with 10 mM dbcAMP and 1 mM IBMX for wells with ST CAP sperm; 56 mM glucose TYH medium with DMSO (vehicle) for wells with SER NC sperm; 56 mM glucose TYH medium with 10 mM dbcAMP and 1 mM IBMX for wells with SER CAP sperm).

Supplementary figure 2

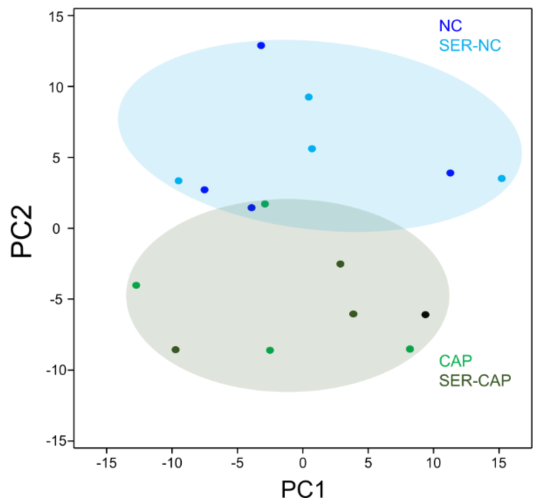

Supplementary Figure 2. PCA scores plot of metabolite profiles generated by 1D ^{1}H NMR spectra, were data from starved sperm were not taken into account. To show the different data clusters, NC and SER-NC sperm values are surrounded by a light blue circle (N=4); CAP and SER-CAP values are surrounded by a light green circle (N=4).

Supplementary figure 3

A

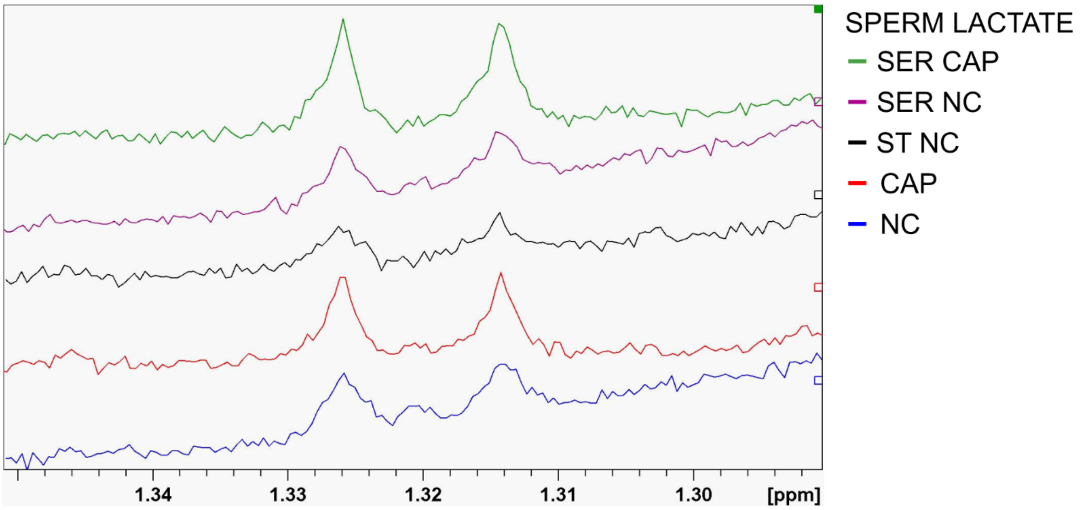

B

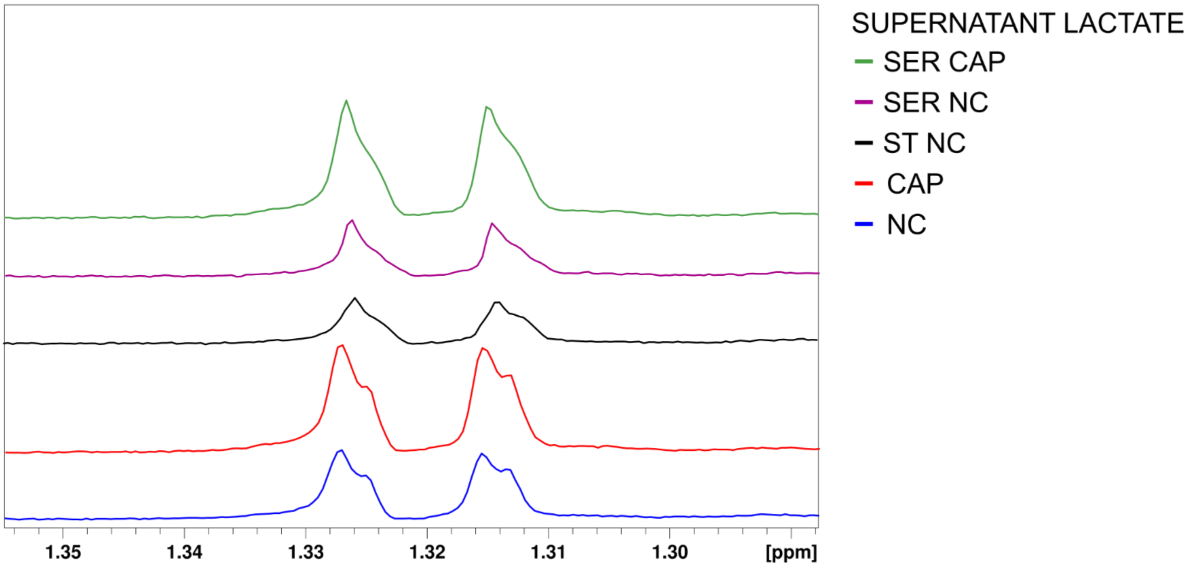

Supplementary Figure 3. Measurement of metabolites by NMR after incubation in the following conditions: continuously incubated with glucose in NC (without BSA and without HCO_3^-) or CAP (with dbcAMP and IBMX); continuously incubated without glucose in NC (ST-NC); incubated without glucose in NC condition until motility stopped (~ 30 to 40 min) and recovered with glucose in non-capacitated medium (SER-NC) or in capacitated medium (SER-CAP). **(A)** Sperm lactate peaks by one-dimensional (1D) ^{1}H-NMR. **(B)** Supernatant lactate peaks by one-dimensional (1D) ^{1}H-NMR.

Supplementary figure 4

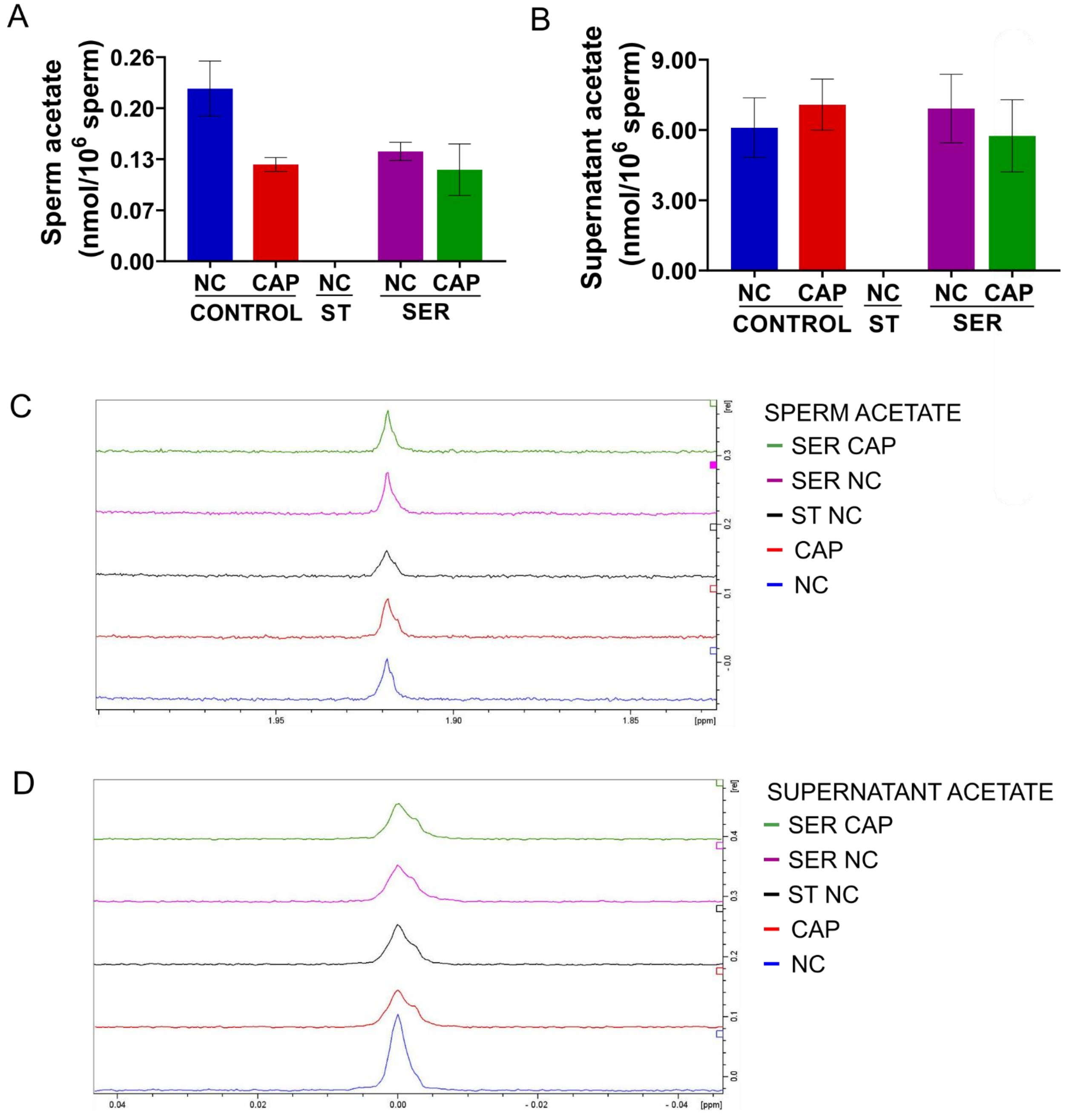

Supplementary Figure 4. Measurement of metabolites by NMR after incubation in the following conditions: continuously incubated with glucose in NC (without BSA and without HCO_3^-) or CAP (with dbcAMP and IBMX); continuously incubated without glucose in NC (ST-NC); incubated without glucose in NC condition until motility stopped (~ 30 to 40 min) and recovered with glucose in non-capacitated medium (SER-NC) or in capacitated medium (SER-CAP). **(A)** Sperm acetate amount determined by 2D NMR ^{1}H-^{13}C HSQC experiments. Results are expressed as the mean ± SEM of 3 independent experiments. Acetate was undetectable in NC ST sperm. T-tests between NC and CAP (control and SER) conditions were performed. **(B)** Supernatant acetate amount determined by 2D NMR ^{1}H-^{13}C HSQC experiments. Results are expressed as the mean ± SEM of 3 independent experiments. Acetate was undetectable in NC ST sperm. T-tests between NC and CAP (control and SER) conditions were performed. **(C)** Sperm acetate peaks by one-dimensional (1D) ^{1}H-NMR. **(D)** Supernatant acetate peaks by one-dimensional (1D) ^{1}H-NMR.

Supplementary figure 5

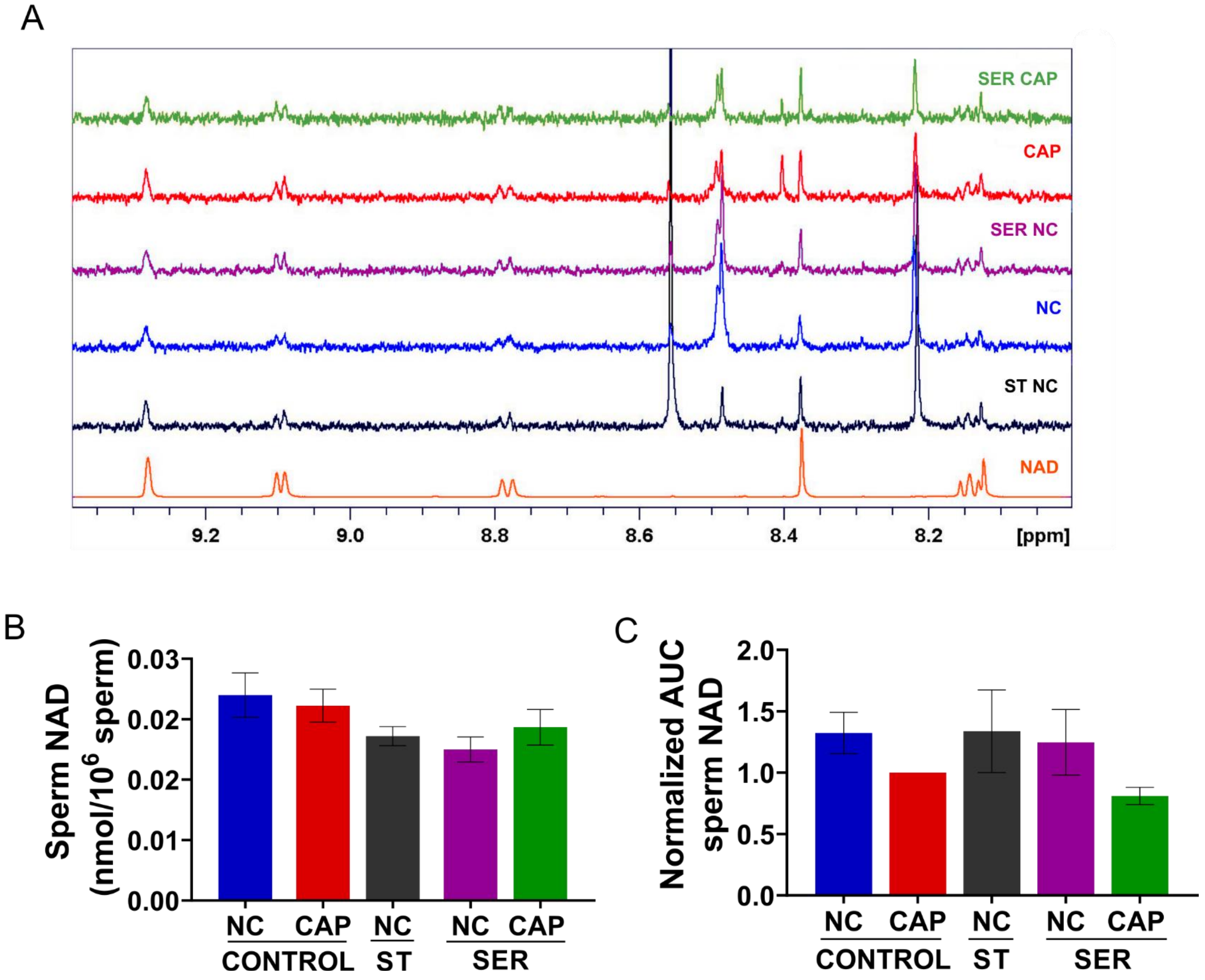

Supplementary Figure 5. Sperm metabolites were determined by NMR and MS after incubation in the following conditions: continuously incubated with glucose in NC (without BSA and without HCO_3^-) or CAP (with dbcAMP and IBMX); continuously incubated without glucose in NC (ST-NC); incubated without glucose in NC condition until motility stopped (~ 30 to 40 min) and recovered with glucose in non-capacitated medium (SER-NC) or in capacitated medium (SER-CAP). **(A)** Sperm Nicotinamide adenine dinucleotide (NAD) peaks by one-dimensional (1D) ^{1}H-NMR. **(B)** Sperm NAD amount determined by 1D NMR ^{1}H experiments. Results are expressed as the mean ± SEM of 4 independent experiments. An Anova was performed. **(C)** Area under the curve (AUC) of NAD MS peak of sperm incubated in the different conditions, normalized to CONTROL CAP. Results are expressed as the mean ± SEM of 7 independent experiments. An Anova with Friedman test and Dunn’s multiple comparisons test was performed.

Supplementary figure 6

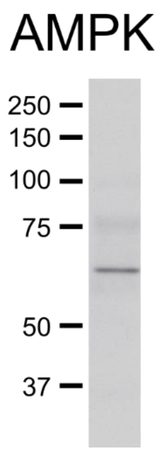

Supplementary Figure 6. **AMPK detection by Western blotting.** Mouse sperm proteins were extracted and separated by 8% SDS-PAGE and immunoblotted using anti-AMPK antibody.
